# Contribution of Frailty to Multimorbidity Patterns and Trajectories: Longitudinal Dynamic Cohort Study of Aging People

**DOI:** 10.2196/45848

**Published:** 2023-06-27

**Authors:** Lucía A Carrasco-Ribelles, Margarita Cabrera-Bean, Marc Danés-Castells, Edurne Zabaleta-del-Olmo, Albert Roso-Llorach, Concepción Violán

**Affiliations:** 1 Fundació Institut Universitari per a la recerca a l’Atenció Primària de Salut Jordi Gol I Gurina (IDIAPJGol) Barcelona Spain; 2 Signal Processing and Communications Group (SPCOM) Department of Signal Theory and Communications Universitat Politècnica de Catalunya (UPC) Barcelona Spain; 3 Unitat de Suport a la Recerca Metropolitana Nord Fundació Institut Universitari per a la recerca a l’Atenció Primària de Salut Jordi Gol I Gurina (IDIAPJGol) Mataró Spain; 4 Grup de REcerca en Impacte de les Malalties Cròniques i les seves Trajectòries (GRIMTRA) (2021 SGR 01537) Fundació Institut Universitari per a la recerca a l’Atenció Primària de Salut Jordi Gol I Gurina (IDIAPJGol) Barcelona Spain; 5 Network for Research on Chronicity, Primary Care, and Health Promotion (RICAPPS) (RD21/0016/0029) Instituto de Salud Carlos III Madrid Spain; 6 Gerència Territorial de Barcelona Institut Català de la Salut Barcelona Spain; 7 Nursing Department Faculty of Nursing Universitat de Girona Girona Spain; 8 Universitat Autònoma de Barcelona Cerdanyola del Vallès Spain; 9 Fundació Institut d'Investigació en ciències de la Salut Germans Trias i Pujol (IGTP) Badalona Spain

**Keywords:** multimorbidity, frailty, clustering, electronic health record, primary care, trajectory

## Abstract

**Background:**

Multimorbidity and frailty are characteristics of aging that need individualized evaluation, and there is a 2-way causal relationship between them. Thus, considering frailty in analyses of multimorbidity is important for tailoring social and health care to the specific needs of older people.

**Objective:**

This study aimed to assess how the inclusion of frailty contributes to identifying and characterizing multimorbidity patterns in people aged 65 years or older.

**Methods:**

Longitudinal data were drawn from electronic health records through the SIDIAP (Sistema d’Informació pel Desenvolupament de la Investigació a l’Atenció Primària) primary care database for the population aged 65 years or older from 2010 to 2019 in Catalonia, Spain. Frailty and multimorbidity were measured annually using validated tools (eFRAGICAP, a cumulative deficit model; and Swedish National Study of Aging and Care in Kungsholmen [SNAC-K], respectively). Two sets of 11 multimorbidity patterns were obtained using fuzzy c-means. Both considered the chronic conditions of the participants. In addition, one set included age, and the other included frailty. Cox models were used to test their associations with death, nursing home admission, and home care need. Trajectories were defined as the evolution of the patterns over the follow-up period.

**Results:**

The study included 1,456,052 unique participants (mean follow-up of 7.0 years). Most patterns were similar in both sets in terms of the most prevalent conditions. However, the patterns that considered frailty were better for identifying the population whose main conditions imposed limitations on daily life, with a higher prevalence of frail individuals in patterns like *chronic ulcers &* *peripheral vascular*. This set also included a dementia-specific pattern and showed a better fit with the risk of nursing home admission and home care need. On the other hand, the risk of death had a better fit with the set of patterns that did not include frailty. The change in patterns when considering frailty also led to a change in trajectories. On average, participants were in 1.8 patterns during their follow-up, while 45.1% (656,778/1,456,052) remained in the same pattern.

**Conclusions:**

Our results suggest that frailty should be considered in addition to chronic diseases when studying multimorbidity patterns in older adults. Multimorbidity patterns and trajectories can help to identify patients with specific needs. The patterns that considered frailty were better for identifying the risk of certain age-related outcomes, such as nursing home admission or home care need, while those considering age were better for identifying the risk of death. Clinical and social intervention guidelines and resource planning can be tailored based on the prevalence of these patterns and trajectories.

## Introduction

Aging is associated with the development of complex conditions, such as multimorbidity and frailty [1,2], which need to be assessed at the individual level. Frailty is a holistic state defined by the biological age-related loss of homeostasis and resistance to stressors, not by particular conditions, and it increases vulnerability to adverse outcomes [2-5]. Frailty can be measured either through the frailty phenotype [5] or cumulative deficit models that consider physical, psychological, and social domains [2,6,7]. On the other hand, multimorbidity is defined as the simultaneous presence of two or more chronic diseases [8]. There is a 2-way causal relationship between multimorbidity and frailty [4,9], and both are associated with higher health care utilization and expenditure [10-14]. Health systems thus need to characterize and monitor the older population to estimate health care and social resource demand.

Electronic health records (EHRs) are essential nowadays to monitor and evaluate patients [15]. Real-world studies use EHRs to obtain a large quantity of observational data from diverse populations. These data sources allow studies to be conducted at a lower cost than traditional epidemiological studies or randomized clinical trials [16,17] and can achieve similar results to randomized clinical trials [18]. Most multimorbidity studies in recent years have used EHRs [19], while EHR-based tools have also been recently developed to measure frailty [20,21].

Clustering is an unsupervised exploratory data analysis technique used for identifying and characterizing population groups. It has already been extensively used to find subgroups of people based on the similarity, in terms of co-occurrence, of their concurrent chronic disease [22-28]. Several systematic reviews [27-29] describe different clustering techniques used to group patients based on multimorbidity, including hierarchical clustering, exploratory factor analysis, multiple correspondence analysis, network analysis, and k-means. K-means and fuzzy c-means are the most common approaches [22-26]. K-means is a hard clustering algorithm that forces each record to belong to a single cluster, while fuzzy c-means is a soft clustering technique that allows records to be simultaneously assigned to multiple clusters through membership probability [30]. In our study, this fuzziness allowed individuals to belong to several clusters, thus creating clusters characterized by broader disease combinations. These techniques have also been applied to identify subgroups of people based on their frailty [31,32]. Although multimorbidity and frailty are strongly associated [4], our review identified only 1 study that considers both simultaneously to build clustering-based patterns [33]. Other authors have related multimorbidity clusters to frailty-related outcomes using regression models [10,26]. However, to our knowledge, no study has assessed whether frailty may influence well-established multimorbidity patterns.

Considering frailty in analyses of multimorbidity is important for tailoring health and social care to the specific needs of the ever-expanding population of elderly people [34]. Moreover, frailty and multimorbidity evolve as people age, and these patterns can change over time, defining a trajectory. Only a few studies have been found that explored these trajectories [19], using hidden Markov models [23,35], latent class growth analysis [36], and descriptive statistics [37]. Furthermore, identifying changes in multimorbidity patterns and trajectories when considering frailty can enrich our understanding of patients’ complex care needs and inform social and health care service strategies [38]. Therefore, our primary aim was to assess how the inclusion of frailty contributes to identifying and characterizing multimorbidity patterns in people aged 65 years or older. Moreover, we described the trajectory of the multimorbidity patterns of individuals as they aged.

## Methods

### Study Design, Setting, Data Source, and Participants

This observational study followed a dynamic cohort from primary care services in Catalonia (Spain) from January 1, 2010, to December 31, 2019. The cohort was drawn from the Information System for Performing Primary Care Research (SIDIAP [Sistema d’Informació pel Desenvolupament de la Investigació a l’Atenció Primària]) database [39]. The SIDIAP database collects pseudoanonymized EHRs from 328 primary care centers in Catalonia managed by the Catalan Health Institute (CHI) since 2005, and it currently has EHRs on more than 8 million patients. This represents almost 80% of the Catalan population and is a reliable representation of the region in terms of age, sex, and geographic distribution [40].

Participants were included at baseline if they were aged 65 years or older in 2010, or were added over the study period as they turned 65 years or arrived in the catchment area (if already aged 65 years or older). They were followed until death, transfer out of the catchment area (lost to follow-up), or end of the study (December 31, 2019). Individuals with no available information, those who did not attend a primary care center over the study period, and those who were aged 100 years or older in 2010 were excluded. Of the initial sample of 1,702,062 individuals, 1,456,052 were finally included.

The CHI linked primary care data with hospital admission data from public health care providers to maintain the pseudoanonymization of the data for researchers. Data included (1) sociodemographic information (ie, sex, age, and socioeconomic status [41]), (2) visits to primary care (ie, date of visit, health professional, and institution visited), (3) clinical measures (eg, BMI, blood pressure, frailty, and dependency questionnaires), (4) all diagnoses made in primary care (using International Classification of Diseases, 10th revision [ICD-10]), (5) laboratory results (eg, cholesterol and glycated hemoglobin), (6) emergency admission episodes (ie, date, number of diagnoses at admission, and length of stay), (7) medications dispensed in pharmacies (using Anatomical Therapeutic Classification [ATC] 5th level), and (8) inclusion in social assistance programs. Socioeconomic status was analyzed by census tract according to a 5-category classification, which considers 22 indicators, for instance, the proportion of the population with a manual occupation or dependency, households without internet access, and single-parent households.

This study complied with the RECORD (Reporting of Studies Conducted using Observational Routinely-collected Data) statement [42] (Multimedia Appendix 1).

### Measurement of Multimorbidity and Frailty

Multimorbidity was measured using the operational definition of the Swedish National Study of Aging and Care in Kungsholmen (SNAC-K), which defined 60 categories of chronic conditions using more than 900 ICD-10 codes, along with clinical, laboratory, and drug-related parameters for assessing certain conditions [43]. The SNAC-K definition of multimorbidity is widely used in studies on older populations, so our results are amenable to comparisons with other studies. Frailty was measured using eFRAGICAP, a validated tool that uses EHRs from Catalan primary care centers [21]. This index considers 36 possible deficits that can be extracted from the EHRs, with 20 related to diseases and 16 related to signs, symptoms, laboratory results, and disabilities. According to the proportion of deficits a person has, their frailty status can be obtained using the cutoff points proposed by Clegg et al [20] (ie, fit, *<*0.12; mild, 0.12-0.24; moderate, 0.24-0.36; and severe ≥0.36). The complete list of codes considered in both multimorbidity and frailty definitions can be found in [43] and [21], respectively.

### Statistical Analysis

Following study approval, data were obtained from SIDIAP. All authors had access to the database. Diagnoses with inconsistent dates and wrong sex-specific diagnoses were excluded. Duplicated diagnoses and clinical measures (same person, same day, and same code) were also excluded. The presence of each of the 60 disease groups and 36 deficits was calculated annually for each participant, according to which conditions were active and which laboratory results or clinical measures were out of range in the participants’ EHRs [21,43]. There were no missing values related to diagnoses or frailty, as a lack of information was interpreted as the absence of the condition or frailty deficit, not as a loss of information. Continuous variables were described using medians and IQRs, as testing for normality showed a nonparametric distribution in all cases, and categorical variables were expressed as absolute and relative frequencies. Clustering and Cox regressions were performed on R v4.1 (R Project for Statistical Computing). Statistical significance was defined as *P<.*05 (2-sided).

### Clustering Analysis

In this study, the information for each included person and year was used to group people based on the similarity of their combined concurrent chronic diseases. Each individual in the clustering analysis contributed records for each year they were included in the study. These groups represented multimorbidity patterns and were found using fuzzy c-means and 2 sets of data. Fuzzy c-means is a fuzzy form of clustering in which records for each individual can be assigned to more than one cluster, or multimorbidity pattern, through fuzzy membership, allowing the pattern definition to be more diverse. Both sets considered chronic conditions, as defined by SNAC-K; however, *multimorbidity & age* also included the age associated with the record, while *multimorbidity & frailty* considered the number of frailty deficits. A detailed description of the clustering analysis can be found in Multimedia Appendix 2 [21,43-47].

Dimensionality was reduced before clustering to simultaneously reduce computational cost and obtain more meaningful variables. First, chronic conditions with a low mean annual prevalence (*<*2%) were removed. Second, a PCAmix transformation [44], which is a mixture of the well-known Principal Component Analysis (PCA) and Multiple Correspondence Analysis (MCA), was applied, and a dimension reduction was performed using the Karlis-Saporta-Spinaki rule [45]. The choice of both the number of clusters (*k*) and the degree of fuzziness (*m*) was validated between *k* ∈ (2*,* 15) and *m* ∈ (1*.*1*,* 1*.*2*,* 1*.*4*,* 1*.*8), calculating analytical indexes using a subset of 100,000 randomly selected participants and 100 repetitions to account for random initialization of the cluster centroids. In addition to the analytical indexes, the opinion on the clinical usefulness and validity of the different sets of patterns of the research team was also considered to select the final *k*. This approach has been used in other studies [22-24,26].

### Description of the Identified Patterns and Trajectories

To characterize the patterns, each person’s annual record was assigned to the pattern with the highest membership probability. The observed/expected (OE) ratio, that is, the ratio between the condition prevalence in the pattern and the condition prevalence in the overall population, and exclusivity, that is, the ratio between the number of individuals in the pattern with the condition and the total number of individuals in the population with the condition, were calculated (see Multimedia Appendix 2 [21,43-47]). Conditions were considered associated with a specific pattern when the exclusivity was ≥25% or the OE ratio was ≥2. The patterns were named in line with these conditions by consensus within the research team (2 general practitioners, 1 nurse, and 2 statisticians), aiming to maximize their clinical utility and consistency with previous literature. In addition, each pattern was described in terms of age, sex, socioeconomic status, multimorbidity and frailty prevalence, smoking and alcohol intake, and health care service use.

**Figure 1:**
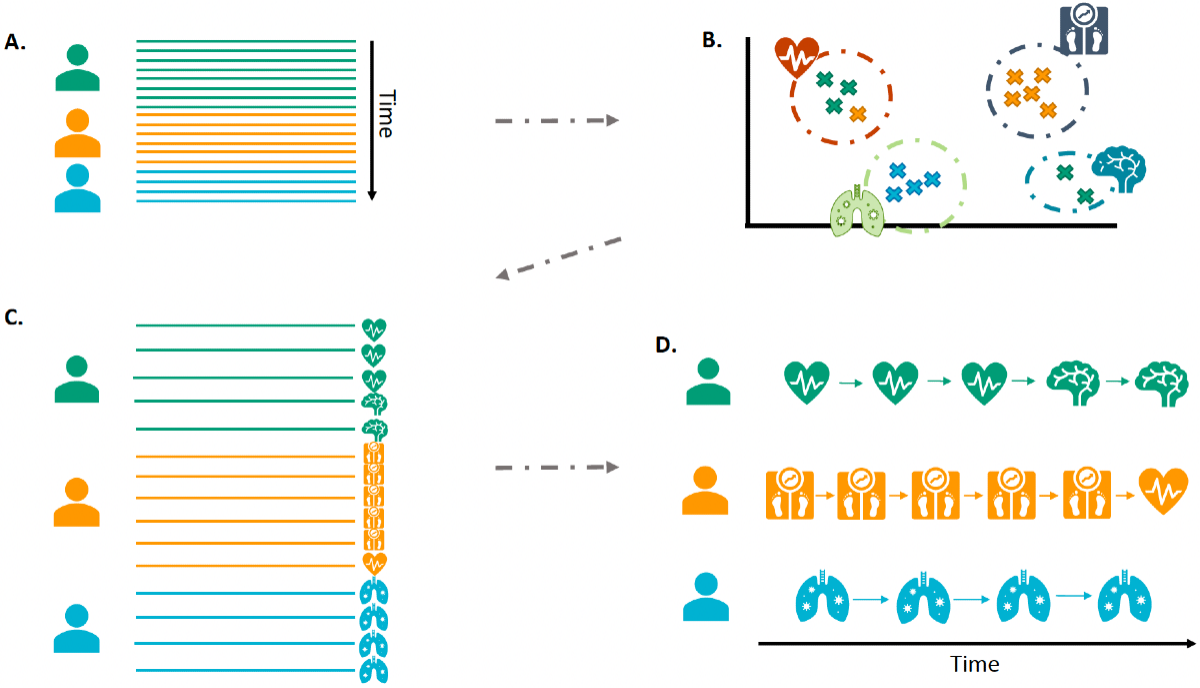
Visual summary of the process of obtaining multimorbidity patterns and trajectories. A: Electronic Health Records (EHR) for three subjects, represented as a line per year of follow-up. B: Clustering gathers each year of each patient. C: Multimorbidity Pattern Assignment. D: Multimorbidity Trajectory.

The clustering model demonstrated the probability of every record and person belonging to each pattern, showing which pattern was most likely for each person each year. Therefore, the evolution among patterns over the study period could be followed as shown in Figure 1. Alluvial and chord plots were used to describe the trajectories, focusing on their evolution with aging, and a transition matrix showed the probability of change from the pattern assigned in the first year of inclusion to that in the last year.

### Cox Regression

The association between the patterns identified for each data set and the following outcomes was calculated: all-cause mortality, nursing home admission, and home care need. The last 2 outcomes were measured using ICD-10 codes (Z59.3 and a Z74 starting code, respectively). The time to event was defined as the interval between cohort entry and the event. Patients were followed until censored (event, lost to follow-up, or end of observation). Cox proportional hazard regression models were fitted to test the association between the patterns and mortality. Similarly, cause-specific Cox models were calculated for nursing home admission and home care need, considering the competing risk of death through a multistate definition. For each model, the Akaike Information Criterion (AIC), *R*^2^, and c-statistic (area under the receiver operating characteristic curve [AUC]) were calculated to assess the goodness of fit, the explained variation, and the predictive capacity of each set of patterns. The only covariate for building these models was the assigned pattern, and was considered time-varying, as each person was assigned to a pattern every year. The proportional hazard assumption was assured in all cases by checking the distribution of the Schoenfeld residuals.

### Ethical Considerations

This study was approved by the Scientific and Ethical Committees of IDIAP (19/518-P) on December 18, 2019. The SIDIAP database is based on optout presumed consent. If a patient decides to opt out, their routine data are excluded from the database. Regarding the hospital admission data, the CHI acts as a trusted third party to execute the linkage and provide the pseudoanonymized data set, without needing informed consent. More information about the management of the SIDIAP database can be found in a previous report [40].

## Results

### Description of the Population

**Figure 2:**
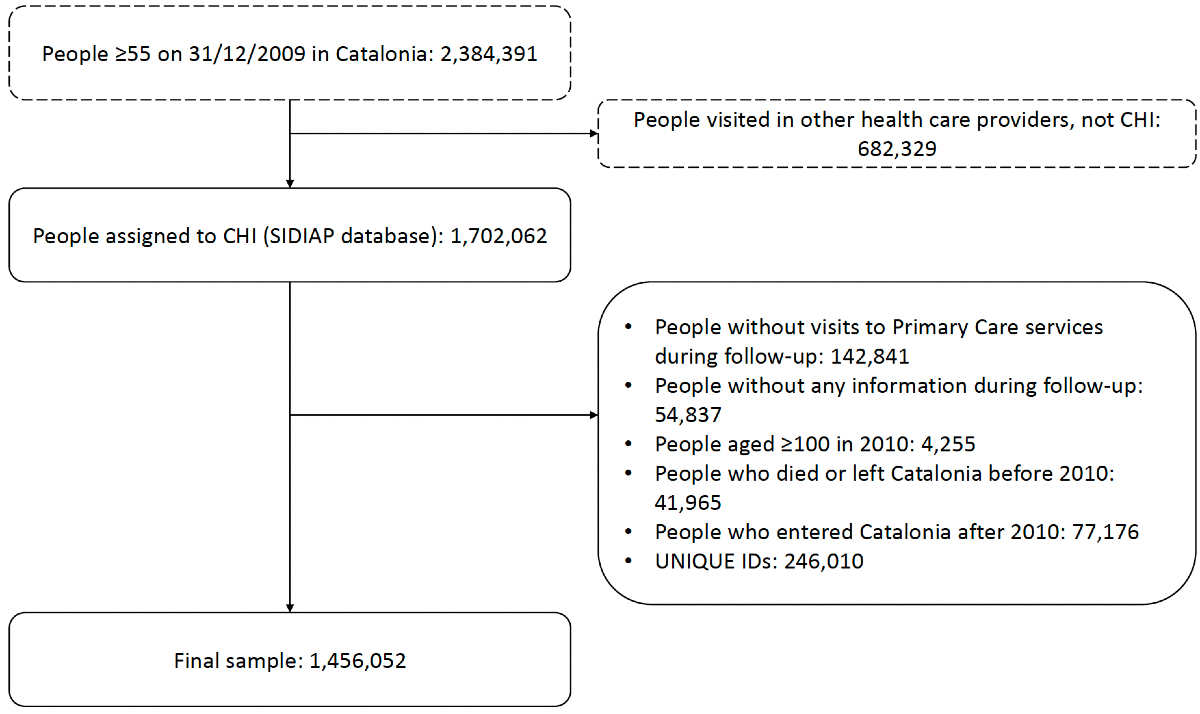
Flow chart of the study population. The figure reports the number of individuals who met each exclusion criterion, as well as the number of individuals who met all the criteria simultaneously (unique IDs). CHI: Catalan Health Institute; SIDIAP: Sistema d’Informació pel Desenvolupament de la Investigació a l’Atenció Primària.

During the follow-up period, 1,456,052 unique participants were included in the study population (Figure 2 and Multimedia Appendix 3), with a mean follow-up of 7.04 (SD 3.15) years. The median age at cohort entry was 69.0 years, and 55.8% (813,074/1,456,052) were women. Most (1,297,810/1,456,052, 89.1%) joined the study with at least two chronic conditions; by the end of follow-up, this proportion was 94.5% (1,376,367/1,456,052). Frailty prevalence increased from 33.4% (486,320/1,456,052) to 60.3% (877,861/1,456,052) (Table 1). The prevalence of each chronic condition is presented in Table S2 in Multimedia Appendix 2.

**Table 1:** Characteristics of the included individuals in their first and last years of follow-up (N=1,456,052).

Variable		First year of follow-up^a,b^	Last year of follow-up^a,b^
Age (years), median (IQR)		69.0 (65.0-77.0)	77.0 (70.0-85.0)
**Sex, n (%)**			
	Women	813,074 (55.8)	813,074 (55.8)
	Men	642,978 (44.2)	642,978 (44.2)
**Deprivation index, n (%)**			
	1 (less deprived)	163,452 (13.2)	163,452 (13.2)
	2	373,120 (30.2)	373,120 (30.2)
	3	411,920 (33.4)	411,920 (33.4)
	4	233,724 (18.9)	233,724 (18.9)
	5 (more deprived)	51,594 (4.2)	51,594 (4.2)
	Missing	222,242	222,242
**Multimorbidity, median (IQR)**			
	SNAC-K^c^ groups of chronic conditions	5.0 (3.0-7.0)	7.0 (5.0-10.0)
**Type of multimorbidity, n (%)**			
	No multimorbidity	158,242 (10.9)	79,685 (5.5)
	2-5 diseases	692,530 (47.6)	401,306 (27.6)
	6-10 diseases	528,865 (36.3)	675,798 (46.4)
	>10 diseases	76,415 (5.3)	299,263 (20.6)
**Frailty, median (IQR)**			
	Deficits	3.0 (2.0-5.0)	6.0 (3.0-9.0)
	Disease-related deficits	1.0 (1.0-2.0)	3.0 (1.0-4.0)
	SSLD^d^ deficits	2.0 (1.0-3.0)	3.0 (1.0-4.0)
**Type of frailty, n (%)**			
	Fit	969,732 (66.6)	578,191 (39.7)
	Mild	404,665 (27.8)	511,505 (35.1)
	Moderate	71,950 (4.9)	263,893 (18.1)
	Severe	9705 (0.7)	102,463 (7.0)
**Smoking status, n (%)**			
	Nonsmoker	845,869 (65.6)	854,407 (61.1)
	Exsmoker	284,398 (22.1)	418,086 (29.9)
	Smoker	159,178 (12.3)	125,421 (9.0)
	Missing	166,607	58,138
**Alcohol intake, n (%)**			
	Nondrinker	412,938 (66.6)	445,245 (66.6)
	Low-risk drinker	195,815 (31.6)	217,360 (32.5)
	High-risk drinker	10,916 (1.8)	6,002 (0.9)
	Missing	836,383	787,445
**Health care service use, median (IQR)**			
	Visits to primary care	9.0 (4.0-16.0)	9.0 (4.0-17.0)
	Distinct drugs^e^	7.0 (3.0-12.0)	7.0 (3.0-11.0)
	Clinical measurements	5.0 (0.0-11.0)	5.0 (0.0-14.0)
	Laboratory measurements	16.0 (0.0-20.0)	16.0 (0.0-24.0)
Receiving home care, n (%)		44,473 (3.1)	194,052 (13.3)
Living in a nursing home, n (%)		31,480 (2.2)	122,844 (8.4)
Death, n (%)		25,254 (1.7)	355,901 (24.4)

^a^Follow-up began in 2010 for individuals aged 65 years or older who were in the catchment area, or the year when they turned 65 years or arrived in the catchment area. The end of follow-up was 2019, the year of death, or the year they left the catchment area. The characteristics were calculated at the end of each year, using the records from that year.

^b^For categorical variables, missing values, if any, are excluded from the calculation of the percentage.

^c^SNAC-K: Swedish National Study of Aging and Care in Kungsholmen.

^d^SSLD: signs, symptoms, laboratory results, and disabilities.

^e^The number of distinct drugs was calculated using the first 5 digits of the Anatomical Therapeutic Classification (ATC) code.

### Description of the Identified Multimorbidity Patterns

Two sets of 11 multimorbidity patterns were identified (*multimorbidity & age* and *multimorbidity & frailty*). The most prevalent chronic conditions in each pattern were similar between sets in most patterns, regardless of whether frailty was included (see Multimedia Appendix 4 for the complete description; a demonstration is shown in Table 2) and the characteristics of their members (see Multimedia Appendix 5 for the complete description; a demonstration is shown in Table 3). The following patterns were identified in both data sets: *allergy & migraine*, *chronic ulcers & peripheral vascular*, *diabetes & obesity*, *genitourinary & respiratory*, *heart & circulatory*, *mental & neurodegenerative*, *neuromusculoskeletal*, *nonspecific*, *peripheral vascular & respiratory*, and *respiratory*. The prevalence of all chronic conditions in *nonspecific* was lower than in the general population. On the other hand, *dementia & motility digestive* appeared only in *multimorbidity & frailty*, while *autoimmune & metabolic* appeared only in *multimorbidity & age*.

Some patterns were female-dominant, such as *allergy & migraine*, *neuromusculoskeletal*, *chronic ulcers & peripheral vascular*, *dementia & motility digestive*, and *mental & neurodegenerative*. On the other hand, *autoimmune & metabolic*, *respiratory*, *genitourinary & respiratory*, and *peripheral vascular & respiratory* were male-dominant, with the latter 2 patterns having the highest rates of smokers and high-risk drinkers. *Allergy & migraine*, *diabetes & obesity*, *genitourinary & respiratory*, *neuromusculoskeletal*, and *nonspecific* included younger individuals, while *chronic ulcers & peripheral vascular*, *dementia & motility digestive*, *heart & circulatory*, and *mental & neurodegenerative* were more common in older individuals. These, together with *peripheral vascular & respiratory*, were the patterns with the highest prevalence of frailty. All patterns, except *nonspecific*, had a high prevalence of multimorbidity.

The emergence of the *dementia & motility digestive* pattern in *multimorbidity & frailty* significantly changed the definition of *mental & neurodegenerative* between sets. As shown in Table 3, in *multimorbidity & frailty*, it comprised younger, less frail individuals with more chronic conditions, such as Parkinson disease and other neurological diseases (ie, Huntington disease or myasthenia), rather than dementia (see Multimedia Appendix 4), while in *multimorbidity & age*, this pattern comprised older and frailer individuals. On the other hand, the *dementia & motility digestive* pattern was made up of older individuals, mostly women, with moderate and severe frailty and a high prevalence of dementia.

**Table 2:** Top 10 conditions in terms of the observed/expected ratio and exclusivity for the heart & circulatory and mental & neurodegenerative patterns.

Pattern and disease			Multimorbidity & frailty					Multimorbidity & age				
			OE^a^ ratio		Exclusivity, %		OE ratio			Exclusivity, %		
**Heart & circulatory**												
	Heart failure	7.86^b^		54.38^b^		7.67^b^			55.57^b^			
	Cardiac valve diseases	7.37^b^		50.98^b^		7.24^b^			52.47^b^			
	Atrial fibrillation	6.54^b^		45.29^b^		6.46^b^			46.81^b^			
	Bradycardia and conduction diseases	5.75^b^		39.83^b^		5.87^b^			42.57^b^			
	Ischemic heart disease	3.69^b^		25.51^b^		3.62^b^			26.22^b^			
	Chronic kidney diseases	2.30^b^		15.95		2.23^b^			16.17			
	Anemia	2.29^b^		15.85		2.21^b^			16.05			
	Cerebrovascular disease	2.16^b^		14.93		2.13^b^			15.41			
	COPD^c^, emphysema, and chronic bronchitis	2.07^b^		14.33		1.99			14.43			
	Inflammatory arthropaties	1.86		12.85		N/A^d^			N/A			
	Chronic pancreas diseases, and biliary tract and gallbladder diseases	N/A		N/A		1.75			12.67			
**Mental & neurodegenerative**												
	Parkinson disease and parkisonism	19.98^b^		90.67^b^		9.24^b^			73.46^b^			
	Other neurological diseases	18.54^b^		84.14^b^		4.68^b^			37.19^b^			
	Dementia	2.24^b^		10.18		6.95^b^			55.24^b^			
	Cerebrovascular disease	2.10^b^		9.53		3.39^b^			26.92^b^			
	Depression and mood diseases	1.76		8.00		2.20^b^			17.50^b^			
	Colitis and related diseases	1.48		6.71		1.83			14.59			
	Anemia	1.38		6.28		2.17^b^			17.26			
	Sleep disorders	1.37		6.22		N/A			N/A			
	Other digestive diseases	1.35		6.11		4.57^b^			36.36^b^			
	Dorsopathies	1.33		6.05		N/A			N/A			
	Chronic kidney diseases	N/A		N/A		1.65			13.10			
	Deafness and hearing impairment	N/A		N/A		1.47			11.69			

^a^OE: observed/expected.

^b^The conditions used to name the pattern. The conditions for the rest of the patterns can be found in Multimedia Appendix 4.

^c^COPD: chronic obstructive pulmonary disease.

^d^N/A: not applicable.

**Table 3:** Description of the participants in the dementia & motility digestive, heart & circulatory, and mental & neurodegenerative patterns.

Variable			Dementia & motility digestive^a,b^		Heart & circulatory^a,b^					Mental & neurodegenerative^a,b^		
			Multimorbidity & frailty (n=783,590)		Multimorbidity & age (n=743,257)		Multimorbidity & frailty (n=709,909)		Multimorbidity & age (n=815,597)			Multimorbidity & frailty (n=465,417)
Age (years), median (IQR)			83.0 (78.0-88.0)		82.0 (77.0-87.0)		82.0 (76.0-87.0)		84.0 (80.0-89.0)			78.0 (72.0-84.0)
**Sex, n (%)**												
	Women	588,734 (75.1)		398,282 (53.6)		382,768 (53.9)		541,039 (66.3)			264,225 (56.8)	
	Men	194,856 (24.9)		344,975 (46.4)		327,141 (46.1)		274,558 (33.7)			201,192 (43.2)	
**Deprivation index, n (%)**												
	1 (less deprived)	109,882 (23.3)		93,677 (20.9)		87,347 (20.3)		119,722 (25.5)			66,020 (21.9)	
	2	95,209 (20.2)		89,449 (20.0)		85,288 (19.8)		97,525 (20.7)			61,623 (20.4)	
	3	99,621 (21.1)		91,379 (20.4)		87,580 (20.3)		99,451 (21.1)			63,731 (21.1)	
	4	87,895 (18.6)		89,469 (20.0)		87,256 (20.3)		83,397 (17.7)			59,089 (19.6)	
	5 (more deprived)	78,990 (16.7)		83,854 (18.7)		82,908 (19.3)		70,225 (14.9)			51,528 (17.1)	
	Missing	311,993		295,429		279,530		345,577			163,426	
**Multimorbidity, median (IQR)**												
	SNAC-K^c^ groups of chronic conditions	9.00 (7.0-11.0)		10.0 (8.0-12.0)		10.0 (8.0-2.0)		8.0 (6.0-10.0)			8.0 (6.0-11.0)	
**Type of multimorbidity, n (%)**												
	No multimorbidity	0 (0.0)		0 (0.0)		0 (0.0)		445 (0.1)			2025 (0.4)	
	2-5 diseases	71,676 (9.2)		45,354 (6.1)		32,702 (4.6)		145,376 (17.8)			80,560 (17.3)	
	6-10 diseases	507,806 (64.8)		395,802 (53.3)		368,236 (51.9)		500,503 (61.4)			254,128 (54.6)	
	>10 diseases	204,108 (26.0)		302,101 (40.6)		308,971 (43.5)		169,273 (20.8)			128,704 (27.7)	
**Frailty, median (IQR)**												
	Deficits	9.0 (7.0-11.0)		9.0 (7.0-11.0)		9.0 (7.0-12.0)		8.0 (6.0-10.0)			7.0 (5.0-10.0)	
	Disease-related deficits	5.0 (3.0-6.0)		3.0 (2.0-5.0)		4.0 (2.0-5.0)		5.0 (3.0-6.0)			3.0 (2.0-5.0)	
	SSLD^d^ deficits	4.0 (3.0-5.0)		5.0 (4.0-7.0)		5.0 (4.0-7.0)		3.0 (2.0-5.0)			4.0 (2.0-5.0)	
**Type of frailty, n (%)**												
	Fit	40,827 (5.21)		49,158 (6.6)		31,624 (4.5)		90,181 (11.1)			96,397 (20.7)	
	Mild	349,212 (44.6)		295,094 (39.7)		268,474 (37.8)		364,281 (44.7)			207,835 (44.7)	
	Moderate	312,751 (39.9)		269,356 (36.2)		276,323 (38.9)		279,797 (34.3)			122,853 (26.4)	
	Severe	80,800 (10.3)		129,649 (17.4)		133,488 (18.8)		81,338 (10.0)			38,332 (8.2)	
**Smoking status, n (%)**												
	Nonsmoker	541,491 (71.8)		454,844 (62.4)		430,558 (61.7)		544,408 (70.5)			299,351 (66.8)	
	Exsmoker	182,092 (24.1)		246,506 (33.8)		240,234 (34.4)		196,319 (25.4)			120,789 (27.0)	
	Smoker	30,528 (4.1)		27,744 (3.8)		27,585 (4.0)		31,545 (4.1)			28,002 (6.2)	
	Missing	29,480		14,163		11,532		43,325			17,275	
**Alcohol intake, n (%)**												
	Nondrinker	315,999 (83.8)		342,552 (75.7)		333,548 (75.6)		297,365 (83.6)			178,703 (76.0)	
	Low-risk drinker	59,668 (15.8)		107,637 (23.8)		104,929 (23.8)		56,921 (16.0)			54,962 (23.4)	
	High-risk drinker	1,596 (0.4)		2,475 (0.6)		2,600 (0.6)		1,218 (0.3)			1,434 (0.6)	
	Missing	406,327		290,593		268,832		460,093			230,318	
**Health care service use, median (IQR)**												
	Visits to primary care	14.0 (8.0-23.0)		20.0 (10.0-33.0)		21.0 (11.0-34.0)		12.0 (6.0-21.0)			13.0 (7.0-21.0)	
	Distinct drugs^e^	10.0 (7.0-14.0)		11.0 (8.0-15.0)		12.0 (8.0-16.0)		9.0 (6.0-13.0)			10.0 (6.0-14.0)	
	Clinical measures	7.00 (2.0-14.0)		11.0 (5.0-19.0)		12.0 (5.0-20.0)		6.0 (1.0-12.0)			7.0 (2.0-14.0)	
	Laboratory results	17.0 (10.0-30.0)		18.0 (10.0-32.0)		18.0 (11.0-33.0)		17.0 (0.0-24.0)			17.0 (3.0-25.0)	

^a^Each column reports the information of all individuals and years included in each pattern. The description of the rest of the patterns can be found in Multimedia Appendix 5.

^b^For categorical variables, missing values, if any, are excluded from the calculation of the percentage.

^c^SNAC-K: Swedish National Study of Aging and Care in Kungsholmen.

^d^SSLD: signs, symptoms, laboratory results, and disabilities.

^e^The number of distinct drugs was calculated using the first 5 digits of the Anatomical Therapeutic Classification (ATC) code.

### Effect of Considering Frailty

Compared to the *multimorbidity & age* patterns, the *multimorbidity & frailty* grouping assigned more frail individuals to the patterns defined by chronic conditions imposing greater limitations on daily life. For example, severe frailty was more prevalent in the *heart & circulatory* pattern and a lack of frailty was more common in the *nonspecific* pattern in *multimorbidity & frailty* than in *multimorbidity & age* (Table 3). In addition, the *dementia & motility digestive* pattern appeared only in *multimorbidity & frailty*, and the definition of *mental & neurodegenerative* changed considerably, as described above.

Each set of patterns behaved differently in terms of the associated outcomes (Table 4). *Multimorbidity & age* patterns had a better goodness of fit (AIC) with mortality than *multimorbidity & frailty*, while *multimorbidity & frailty* patterns had a better or similar goodness of fit with nursing home admission or home care need. A similar behavior was observed for *R*^2^, while both sets of patterns achieved similar results in all outcomes in terms of AUC. Regarding the hazard ratios, they were very similar between *multimorbidity & age* and *multimorbidity & frailty* in all patterns and outcomes, except *mental & neurodegenerative*, which were lower in *multimorbidity & frailty*. *Chronic ulcers & peripheral vascular*, *dementia & motility digestive*, *heart & circulatory*, *mental & neurodegenerative*, and *peripheral vascular & respiratory* showed the highest risk of death. All patterns had a higher risk of nursing home admission than *nonspecific*, with *dementia & motility digestive*, *chronic ulcers & peripheral vascular*, and *mental & neurodegenerative* standing out. These latter patterns, together with *heart & circulatory*, also had a higher risk of home care need.

**Table 4:** Data for the unadjusted survival models using the multimorbidity patterns as the time-varying covariate.

Pattern^a,b^	Mortality			Nursing home admission			Home care need	
	Multimorbidity & age, HR^c^ (95% CI)	Multimorbidity & frailty, HR (95% CI)	Multimorbidity & age, HR (95% CI)		Multimorbidity & frailty, HR (95% CI)	Multimorbidity & age, HR (95% CI)		Multimorbidity & frailty, HR (95% CI)
Allergy & migraine	0.39 (0.39-0.70)	0.43 (0.42-0.44)	1.68 (1.61-1.76)		1.53 (1.45-1.61)	2.50 (2.41-2.59)		2.98 (2.87-3.10)
Autoimmune & metabolic	0.97 (0.96-0.99)	N/A^d^	2.51 (2.40-2.62)		N/A	4.66 (4.52-4.81)		N/A
Chronic ulcers & peripheral vascular	6.94 (6.85-7.03)	6.87 (6.78-6.96)	22.28 (21.47-23.12)		23.43 (22.58-24.31)	26.25 (25.44-27.08)		34.36 (33.24-35.52)
Dementia & motility digestive	N/A	2.88 (2.86-2.92)	N/A		22.95 (22.25-23.67)	N/A		25.38 (24.67-26.12)
Diabetes & obesity	0.7 (0.69-0.71)	0.62 (0.61-0.63)	1.62 (1.56-1.69)		1.42 (1.37-1.48)	0.68 (0.67-0.69)		3.23 (3.13-3.34)
Genitourinary & respiratory	0.8 (0.79-0.81)	1.05 (1.04-1.07)	1.22 (1.16-1.28)		1.48 (1.42-1.55)	1.69 (1.63-1.75)		2.97 (2.86-3.07)
Heart & circulatory	3.14 (3.10-3.18)	3.07 (3.03-3.10)	7.71 (7.45-7.98)		8.13 (7.86-8.42)	15.64 (15.23-16.07)		21.59 (20.97-22.22)
Mental & neurodegenerative	3.56 (3.52-3.60)	1.96 (1.93-1.99)	24.41 (23.66-25.18)		10.91 (10.53-11.30)	20.46 (19.93-21.00)		16.98 (16.46-17.51)
Neuromusculoskeletal	0.32 (0.31-0.33)	0.31 (0.31-0.32)	1.61 (1.54-1.68)		1.43 (1.36-1.49)	3.36 (3.25-3.46)		3.83 (3.70-3.96)
Peripheral vascular & respiratory	2.04 (2.01-2.07)	1.98 (1.95-2.01)	3.99 (3.82-4.17)		4.02 (3.85-4.20)	7.53 (7.30-7.77)		9.54 (9.23-9.87)
Respiratory	0.9 (0.89-0.92)	0.87 (0.86-0.89)	3.11 (2.98-3.26)		2.98 (2.84-3.12)	5.78 (5.60-5.97)		7.10 (6.86-7.35)
AIC^e^	9,605,078^f^	9,637,872	10,261,814		9,996,581^f^	10,945,802		10,924,083^f^
R^2^	0.37^f^	0.32	0.21		0.38^f^	0.36		0.39^f^
AUC^g^	0.713^f^	0.705	0.718^f^		0.717	0.719		0.721^f^

^a^Nonspecific was used as reference.

^b^All *P*<.001.

^c^HR: hazard ratio.

^d^N/A: not applicable.

^e^AIC: Akaike Information Criterion.

^f^The set of patterns that achieved better performance for each metric and outcome.

^g^AUC: area under the receiver operating characteristic curve.

### Study of the Trajectories

**Figure 3:**
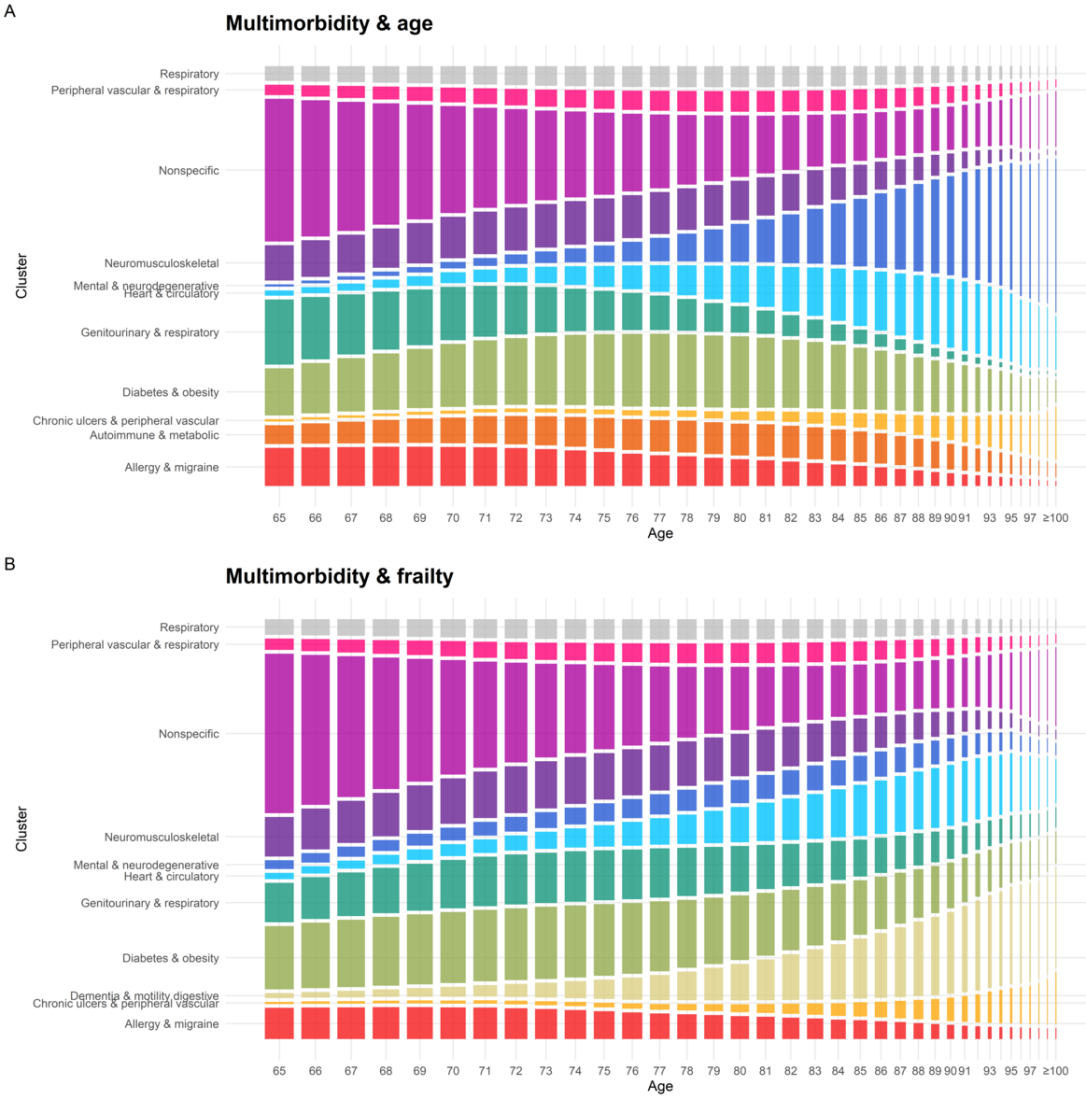
Prevalence of each multimorbidity pattern for each age. (A) Multimorbidity & age; (B) multimorbidity & frailty. For each age, the information considered is from the individuals of that age in any time, regardless of the year of the study.

Over the follow-up, individuals changed patterns an average of 1.75 (*multimorbidity & age*) and 1.85 (*multimorbidity & frailty*) times, while 45.1% (656,778/1,456,052) of the individuals remained in the same pattern. Multimedia Appendix 6 shows that these values depended on the length of the trajectory. For example, people with 5 years of follow-up had an average of 1.55 different patterns in their trajectory, while around 54.5% (52,634/96,578, 54.5% in *multimorbidity & age*; 52,586/96,578, 54.4% in *multimorbidity & frailty*) remained in the same pattern. In those with 10 years of follow-up, these values changed to 2.15 and 26.6%, respectively. The prevalence of most patterns varied with age, as shown in Figure 3. The prevalence of *nonspecific* showed the largest reduction, as most of its members developed diseases as they aged and shifted to more disease-specific patterns. On the other hand, *heart & circulatory* and *mental & neurodegenerative* for *multimorbidity & age*, and *dementia & motility digestive* for *multimorbidity & frailty* showed the highest increases in prevalence with age.

**Figure 4:**
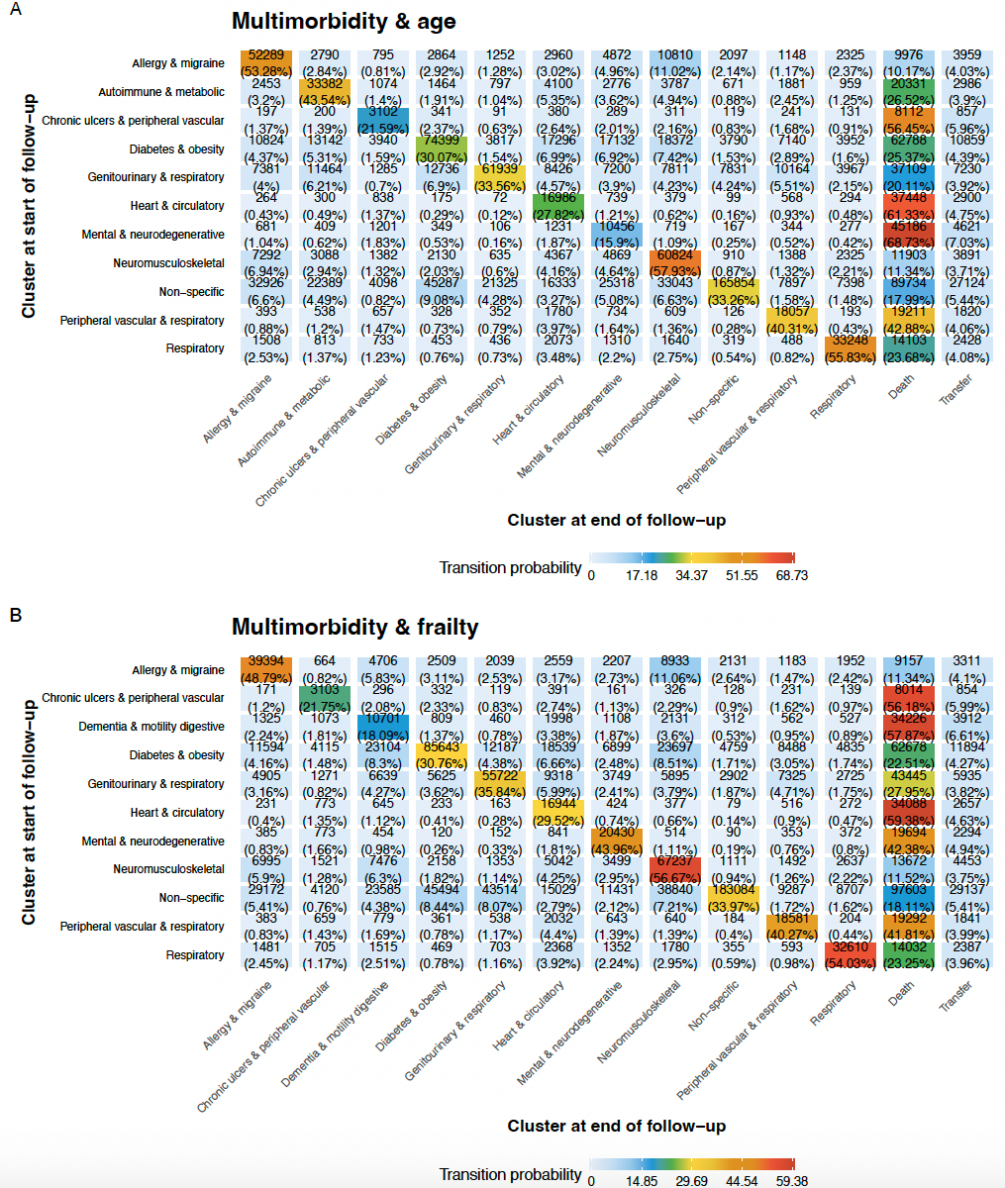
Transition matrices for multimorbidity & age (A) and multimorbidity & frailty (B) with k=11. Each cell shows the proportion of individuals transitioning from their initial pattern (y-axis) to the last pattern observed (x-axis).

Figure 4 shows the transition matrices for both *multimorbidity & age* and *multimorbidity & frailty*. *Chronic ulcers & peripheral vascular*, *heart & circulatory*, *mental & neurodegenerative*, and *dementia & motility digestive* were the multimorbidity patterns most closely associated with mortality, as more than 55% of the individuals who started in them died during follow-up (Figure 4). On the other hand, the *allergy & migraine*, *neuromusculoskeletal*, and *nonspecific* clusters showed the lowest mortality. When considering frailty, the number of individuals transitioning to *mental & neurodegenerative* from any pattern was reduced. Nevertheless, considering the general stability of the trajectories, a relatively high percentage of individuals transitioned to *dementia & motility digestive*. Multimedia Appendix 7 (*multimorbidity & age*) and Multimedia Appendix 8 (*multimorbidity & frailty*) show the evolution of 3 subsets of 50 random individuals aged 65, 75, and 85 years, respectively, in 2010 to illustrate how multimorbidity trajectories vary with age. These figures show that the percentage of individuals starting in *nonspecific* decreased inversely with age at cohort entry, as did the percentage of individuals remaining in this pattern throughout follow-up.

## Discussion

This study aimed to assess how frailty contributes to the characterization of multimorbidity patterns, as identified through clustering techniques. In a Mediterranean cohort of 1,456,052 people aged over 65 years, 2 sets of 11 multimorbidity patterns were identified based on the presence of chronic conditions. One considered age and the other considered the number of frailty deficits. The consideration of frailty modified multimorbidity patterns, revealing patterns with better goodness of fit to the outcomes related to frail aging, such as nursing home admission and home care need, and gathering more frail individuals in patters characterized by more limiting conditions. The better fit to aging-related outcomes when considering frailty has been previously reported [48]. Moreover, the trajectories of multimorbidity patterns were different when considering frailty. When considering only conditions and age, and not frailty, the patterns showed better goodness of fit with the outcome of death, and described an additional disease-related pattern.

When considering frailty, an additional pattern more specific to aging was identified (*dementia & motility digestive*). Distinguishing this pattern from *mental & neurodegenerative* can enable more personalized clinical treatment. In the former case, palliative care would be appropriate, while the latter would call for active treatments to delay disease progression. Patterns defined by less limiting conditions for daily life, such as *diabetes & obesity*, had a lower prevalence of frailty when frailty was considered, in contrast with more limiting patterns such as *heart & circulatory*. For this same reason, the *genitourinary & respiratory* pattern also had a higher proportion of men when frailty was considered, as it included more individuals with prostate diseases, which usually lead to more frailty [49,50], while the same pattern included more women when frailty was not considered, as it involved addictions (ie, drug, alcohol, or tobacco use), which usually involve less frailty than prostate diseases.

Most of the 11 multimorbidity patterns were similarly described whether frailty was considered or not and could be classified into concordant or discordant multimorbidity patterns depending on whether the conditions defining the pattern shared pathophysiology or approaches to clinical management [51,52]. For example, *heart & circulatory*, *chronic ulcers & peripheral vascular*, *respiratory*, and *diabetes & obesity* are patterns of concordant multimorbidity, while *genitourinary & respiratory* and *peripheral vascular & respiratory* are patterns of discordant multimorbidity. Discordant conditions might be grouped together because of shared risk factors, such as smoking or alcohol intake, rather than shared pathophysiology. This is the case for *genitourinary & respiratory* and *peripheral vascular & respiratory*, which mainly included men, with a higher prevalence of smokers and high-risk drinkers. Another risk factor these individuals may share could be their genetics, which can also influence the development of multimorbidity [53]. This discordance makes treatment more complex [52]; thus, identifying patients who follow a discordant multimorbidity pattern can signal a need for integrated care.

Regarding the trajectories of multimorbidity patterns, the *heart & circulatory* and *mental & neurodegenerative* patterns (when not considering frailty), and the *dementia & digestive motility* pattern (when considering frailty) showed the most changes over time. Their prevalence increased the most with aging, and the patients in these groups had the highest probability of transitioning to death at the end of their follow-up. This may be due to the high prevalence of frailty in these patterns, and the association of frailty with death [54]. The *nonspecific* pattern also had a high probability of transition, as it included healthier individuals who transitioned to other patterns as diseases appeared with age. *Peripheral vascular & respiratory* involved a burden of heart conditions, which could lead the trajectory toward *heart & circulatory*. However, many of these individuals died, possibly before the onset of the cardiac diseases could be recorded in the EHRs. On the other hand, *neuromusculoskeletal* and *respiratory* were the patterns with the fewest transitions during follow-up, with more than 50% of the individuals remaining in the same pattern throughout the study period. This may be because both patterns tended to evolve toward disability rather than death or the development of other comorbidities, and nowadays, treatments are more effective in maintaining the status of patients. In our study, trajectories included a mean of 1.8 different multimorbidity patterns. Even people who had 10 years of follow-up did not make 3 changes on average; thus, the trajectories can be considered quite stable. This result is similar to that found in previous research in this same population, albeit with slightly different methods, where 59% of individuals did not change their pattern [23] over 5 years of follow-up (54.5% in this study).

In terms of patient-based multimorbidity patterns reported in the literature, the substantial variability [55] could be attributed to differences in the populations or to the lack of consensus on how many and which diseases should be considered in determining multimorbidity [9]. However, the most commonly reported patterns from multimorbidity data include cardiac, cognitive, psychiatric, musculoskeletal, respiratory, and genitourinary system diseases [27,28], and these patterns have also been found in this work. Few studies have described multimorbidity trajectories [19], and none considered frailty in their definition; thus, our study is pioneering in this line of research. Only 1 scoping review on multimorbidity trajectories compiled evidence from 34 studies, finding significant associations between multimorbidity and adverse outcomes [56]. However, the heterogeneity of the described methods and the long-term conditions considered in each study preclude a robust comparison.

This study has strengths and limitations. We used a large high-quality database [57] along with standardized and validated tools to identify both multimorbidity [43] and frailty [20,21]. EHRs are a representation of real-world data and may, despite cleaning, contain mistakes inherent to daily clinical practice. This could represent an information bias, but SIDIAP has implemented several standardized quality protocols to avoid it [40]. In addition, we considered the absence of any condition or frailty information in the EHRs to indicate an absence of that condition or frailty status in the individual. Therefore, some chronic conditions or frailty deficits could be underreported among patients who visit primary care centers less frequently, constituting an information bias. Consequently, only data available in the EHRs were considered, and to avoid the creation of unreal records, no missing values were imputed. The SIDIAP database is representative of the population of Catalonia [40], so its use does not imply a selection bias. We excluded people who did not visit primary care during the entire study period in order to eliminate those with private health insurance; however, we cannot rule out that some were healthy individuals. After the first filtering, a criterion based on the predominance of diseases in the identified patterns had to be defined and used to make the final selection. The inclusion of all potential diagnoses would have entailed greater complexity, which would have hindered both the interpretation of findings and the comparison with other studies. Other studies have proceeded in the same way [22,23]. Clustering is an unsupervised exploratory technique whose results depend on the population. Therefore, different patterns could be identified from a database in another region. However, the variables used to generate the patterns were obtained using electronic tools based on routine EHR data, such as SNAC-K and eFRAGICAP, so their acquisition is reproducible in information systems in other countries. This increases the international applicability of this study, helping to establish multimorbidity management worldwide. In addition, clustering techniques can suffer from dependency on random initialization, and there is no guarantee of optimal clustering. To minimize this disadvantage, 100 repetitions with different seeds were performed when optimizing the choice of the number of clusters. Particularly in fuzzy clustering techniques, the membership probability in the heuristic global cost function depends on the number of clusters, and specifying a wrong number of clusters may affect the clustering solution [58]. However, we have validated the number of clusters both analytically and clinically.

Care for older people requires holistic patient-centered care plans that are effectively coordinated and minimally disruptive, considering the social and family context in which health care activities are managed, decisions are made, and care is experienced. As a future line of work, these multimorbidity patterns could be used as adjustment covariates in prediction models for outcomes, such as those reported here, or others, such as emergency admission. Similarly, artificial intelligence–based models that predict the timing and direction of transitions between patterns can be developed. These models could help to improve and anticipate decision-making regarding end-of-life management.

More work can be done on the study of trajectories, such as the development of care and treatment guidelines that simultaneously consider the current individual’s multimorbidity pattern and the trajectory over time. Sequence analysis, which combines longitudinal analysis and clustering, could also be used to identify trajectories [59]. The relationship between genetics and concordant and discordant multimorbidity patterns could also be studied.

This study took a person-centered approach, offering relevant information about the multimorbidity patterns and trajectories in the aging population based on age, frailty, and other health determinants. Multimorbidity and frailty can define aging, so both characteristics are relevant considerations made when designing and developing tools, such as multimorbidity patterns, to characterize the aging population. When considering the frailty of individuals, the estimation of outcomes, such as nursing home admission and home care need, improved, as did the characterization of the patterns themselves in terms of how limiting their main chronic conditions can be. The consideration of both multimorbidity and frailty can help to improve treatment guidelines, social assistance, and decision-making in primary care. If most patients in a primary care center follow patterns associated with frailty, increased spending on home-based care services and integrated care programs may be warranted, while a higher burden of multimorbidity would imply higher spending on physician visits. Therefore, and echoing other studies, we recommend that future research involving older populations consider frailty [60].

## Appendix

Multimedia Appendix 1 RECORD (Reporting of Studies Conducted using Observational Routinely-collected Data) statement.

Multimedia Appendix 2 Supplementary methods.

Multimedia Appendix 3 Longitudinal flowchart of included persons during the study period (year 2010-2019).

Multimedia Appendix 4 The top 10 conditions in terms of the observed/expected ratio and exclusivity for each pattern for both multimorbidity & age and multimorbidity & frailty.

Multimedia Appendix 5 Description of the participants gathered in each pattern.

Multimedia Appendix 6 Statistics of the trajectories according to follow-up time.

Multimedia Appendix 7 Trajectories of 50 random participants aged 65, 75, or 85 years in 2010 (baseline year) according to multimorbidity & age patterns.

Multimedia Appendix 8 Trajectories of 50 random participants aged 65, 75, or 85 years in 2010 (baseline year) according to multimorbidity & frailty patterns.

## Abbreviations

AIC: Akaike Information Criterion
ATC: Anatomical Therapeutic Classification
AUC: area under the receiver operating characteristic curve
CHI: Catalan Health Institute
EHR: electronic health record
ICD-10: International Classification of Diseases, 10th revision
OE: observed/expected
SIDIAP: Sistema d’Informació pel Desenvolupament de la Investigació a l’Atenció Primària
SNAC-K: Swedish National Study of Aging and Care in Kungsholmen

## Data Availability

The data used in this study are only available to the participating researchers, in accordance with current European and national laws. Thus, distribution of the data is not allowed. However, researchers from public institutions can request data from SIDIAP. Further information is available online [61].

## References

[1] Barnett K, Mercer S, Norbury M, Watt G, Wyke S, Guthrie B (2012). Epidemiology of multimorbidity and implications for health care, research, and medical education: a cross-sectional study. The Lancet.

[2] Clegg A, Young J, Iliffe S, Rikkert M, Rockwood K (2013). Frailty in elderly people. The Lancet.

[3] Rockwood K, Mitnitski A, Song X, Steen B, Skoog I (2006). Long-term risks of death and institutionalization of elderly people in relation to deficit accumulation at age 70. J Am Geriatr Soc.

[4] Villacampa-Fernández P, Navarro-Pardo E, Tarín JJ, Cano A (2017). Frailty and multimorbidity: Two related yet different concepts. Maturitas.

[5] Fried LP, Tangen CM, Walston J, Newman AB, Hirsch C, Gottdiener J, Seeman T, Tracy R, Kop WJ, Burke G, McBurnie MA, Cardiovascular Health Study Collaborative Research Group (2001). Frailty in older adults: evidence for a phenotype. J Gerontol A Biol Sci Med Sci.

[6] Mitnitski A, Mogilner A, Rockwood K (2001). Accumulation of deficits as a proxy measure of aging. ScientificWorldJournal.

[7] Rodríguez-Mañas L, Féart C, Mann G, Viña J, Chatterji S, Chodzko-Zajko W, Gonzalez-Colaço Harmand M, Bergman H, Carcaillon L, Nicholson C, Scuteri A, Sinclair A, Pelaez M, Van der Cammen T, Beland F, Bickenbach J, Delamarche P, Ferrucci L, Fried LP, Gutiérrez-Robledo LM, Rockwood K, Rodríguez Artalejo F, Serviddio G, Vega E, FOD-CC group (Appendix 1) (2013). Searching for an operational definition of frailty: a Delphi method based consensus statement: the frailty operative definition-consensus conference project. J Gerontol A Biol Sci Med Sci.

[8] (2018). Multimorbidity: a priority for global health research. The Academy of Medical Sciences.

[9] Vetrano D, Palmer K, Marengoni A, Marzetti E, Lattanzio F, Roller-Wirnsberger R, Lopez Samaniego L, Rodríguez-Mañas L, Bernabei R, Onder G, Joint Action ADVANTAGE WP4 Group (2019). Frailty and Multimorbidity: A Systematic Review and Meta-analysis. J Gerontol A Biol Sci Med Sci.

[10] Marengoni A, Angleman S, Melis R, Mangialasche F, Karp A, Garmen A, Meinow B, Fratiglioni L (2011). Aging with multimorbidity: a systematic review of the literature. Ageing Res Rev.

[11] Librero J, Peiró S, Ordiñana R (1999). Chronic comorbidity and outcomes of hospital care: length of stay, mortality, and readmission at 30 and 365 days. J Clin Epidemiol.

[12] Roe L, Normand C, Wren M, Browne J, O'Halloran AM (2017). The impact of frailty on healthcare utilisation in Ireland: evidence from the Irish longitudinal study on ageing. BMC Geriatr.

[13] Han L, Clegg A, Doran T, Fraser L (2019). The impact of frailty on healthcare resource use: a longitudinal analysis using the Clinical Practice Research Datalink in England. Age Ageing.

[14] García-Nogueras I, Aranda-Reneo I, Peña-Longobardo LM, Oliva-Moreno J, Abizanda P (2017). Use of Health Resources and Healthcare Costs associated with Frailty: The FRADEA Study. J Nutr Health Aging.

[15] Zhang J, Symons J, Agapow P, Teo J, Paxton C, Abdi J, Mattie H, Davie C, Torres AZ, Folarin A, Sood H, Celi LA, Halamka J, Eapen S, Budhdeo S (2022). Best practices in the real-world data life cycle. PLOS Digit Health.

[16] Blonde L, Khunti K, Harris SB, Meizinger C, Skolnik NS (2018). Interpretation and Impact of Real-World Clinical Data for the Practicing Clinician. Adv Ther.

[17] Ho Y, Hu F, Lee P (2020). The Advantages and Challenges of Using Real-World Data for Patient Care. Clin Transl Sci.

[18] Wang S, Schneeweiss S, Franklin JM, Desai RJ, Feldman W, Garry EM, Glynn RJ, Lin KJ, Paik J, Patorno E, Suissa S, D'Andrea E, Jawaid D, Lee H, Pawar A, Sreedhara SK, Tesfaye H, Bessette LG, Zabotka L, Lee SB, Gautam N, York C, Zakoul H, Concato J, Martin D, Paraoan D, Quinto K, RCT-DUPLICATE Initiative (2023). Emulation of Randomized Clinical Trials With Nonrandomized Database Analyses: Results of 32 Clinical Trials. JAMA.

[19] Ho I, Azcoaga-Lorenzo A, Akbari A, Black C, Davies J, Hodgins P, Khunti K, Kadam U, Lyons R, McCowan C, Mercer S, Nirantharakumar K, Guthrie B (2021). Examining variation in the measurement of multimorbidity in research: a systematic review of 566 studies. The Lancet Public Health.

[20] Clegg A, Bates C, Young J, Ryan R, Nichols L, Ann Teale E, Mohammed MA, Parry J, Marshall T (2016). Development and validation of an electronic frailty index using routine primary care electronic health record data. Age Ageing.

[21] Orfila F, Carrasco-Ribelles L, Abellana R, Roso-Llorach A, Cegri F, Reyes C, Violán C (2022). Validation of an electronic frailty index with electronic health records: eFRAGICAP index. BMC Geriatr.

[22] Violán C, Foguet-Boreu Q, Fernández-Bertolín S, Guisado-Clavero M, Cabrera-Bean M, Formiga F, Valderas JM, Roso-Llorach A (2019). Soft clustering using real-world data for the identification of multimorbidity patterns in an elderly population: cross-sectional study in a Mediterranean population. BMJ Open.

[23] Violán C, Fernández-Bertolín S, Guisado-Clavero M, Foguet-Boreu Q, Valderas J, Vidal Manzano J, Roso-Llorach A, Cabrera-Bean M (2020). Five-year trajectories of multimorbidity patterns in an elderly Mediterranean population using Hidden Markov Models. Sci Rep.

[24] Guisado-Clavero M, Roso-Llorach A, López-Jimenez T, Pons-Vigués M, Foguet-Boreu Q, Muñoz MA, Violán C (2018). Multimorbidity patterns in the elderly: a prospective cohort study with cluster analysis. BMC Geriatr.

[25] Marengoni A, Akugizibwe R, Vetrano D, Roso-Llorach A, Onder G, Welmer A, Calderón-Larrañaga A (2021). Patterns of multimorbidity and risk of disability in community-dwelling older persons. Aging Clin Exp Res.

[26] Tazzeo C, Rizzuto D, Calderón-Larrañaga A, Roso-Llorach A, Marengoni A, Welmer A, Onder G, Trevisan C, Vetrano DL (2021). Multimorbidity patterns and risk of frailty in older community-dwelling adults: a population-based cohort study. Age Ageing.

[27] Prados-Torres A, Calderón-Larrañaga A, Hancco-Saavedra J, Poblador-Plou B, van den Akker M (2014). Multimorbidity patterns: a systematic review. J Clin Epidemiol.

[28] Violan C, Foguet-Boreu Q, Flores-Mateo G, Salisbury C, Blom J, Freitag M, Glynn L, Muth C, Valderas JM (2014). Prevalence, determinants and patterns of multimorbidity in primary care: a systematic review of observational studies. PLoS One.

[29] Ng S, Tawiah R, Sawyer M, Scuffham P (2018). Patterns of multimorbid health conditions: a systematic review of analytical methods and comparison analysis. Int J Epidemiol.

[30] Halkidi M, Batistakis Y, Vazirgiannis M (2001). On Clustering Validation Techniques. Journal of Intelligent Information Systems.

[31] Ward R, Orkaby A, Dumontier C, Charest B, Hawley C, Yaksic E, Quach L, Kim DH, Gagnon DR, Gaziano JM, Cho K, Djousse L, Driver JA (2021). Trajectories of Frailty in the 5 Years Prior to Death Among U.S. Veterans Born 1927-1934. J Gerontol A Biol Sci Med Sci.

[32] Zamora-Sánchez JJ, Zabaleta-Del-Olmo E, Fernández-Bertolín S, Gea-Caballero V, Julián-Rochina I, Pérez-Tortajada G, Amblàs-Novellas J (2021). Profiles of Frailty among Older People Users of a Home-Based Primary Care Service in an Urban Area of Barcelona (Spain): An Observational Study and Cluster Analysis. J Clin Med.

[33] Bekić S, Babič F, Filipčić I, Trtica Majnarić L (2019). Clustering of Mental and Physical Comorbidity and the Risk of Frailty in Patients Aged 60 Years or More in Primary Care. Med Sci Monit.

[34] Foreman K, Marquez N, Dolgert A, Fukutaki K, Fullman N, McGaughey M, Pletcher M, Smith A, Tang K, Yuan C, Brown Jc, Friedman J, He J, Heuton K, Holmberg M, Patel D, Reidy P, Carter A, Cercy K, Chapin A, Douwes-Schultz D, Frank T, Goettsch F, Liu P, Nandakumar V, Reitsma M, Reuter V, Sadat N, Sorensen R, Srinivasan V, Updike R, York H, Lopez A, Lozano R, Lim S, Mokdad A, Vollset S, Murray C (2018). Forecasting life expectancy, years of life lost, and all-cause and cause-specific mortality for 250 causes of death: reference and alternative scenarios for 2016–40 for 195 countries and territories. The Lancet.

[35] Alaeddini A, Jaramillo C, Faruqui S, Pugh M (2018). Mining Major Transitions of Chronic Conditions in Patients with Multiple Chronic Conditions. Methods Inf Med.

[36] Jackson C, Dobson A, Tooth L, Mishra G (2015). Body mass index and socioeconomic position are associated with 9-year trajectories of multimorbidity: A population-based study. Prev Med.

[37] Xu X, Mishra G, Dobson A, Jones M (2018). Progression of diabetes, heart disease, and stroke multimorbidity in middle-aged women: A 20-year cohort study. PLoS Med.

[38] Kastner M, Cardoso R, Lai Y, Treister V, Hamid JS, Hayden L, Wong G, Ivers NM, Liu B, Marr S, Holroyd-Leduc J, Straus SE (2018). Effectiveness of interventions for managing multiple high-burden chronic diseases in older adults: a systematic review and meta-analysis. CMAJ.

[39] SIDIAP.

[40] Recalde M, Rodríguez C, Burn E, Far M, García D, Carrere-Molina J, Benítez M, Moleras A, Pistillo A, Bolíbar B, Aragón M, Duarte-Salles T (2022). Data Resource Profile: The Information System for Research in Primary Care (SIDIAP). Int J Epidemiol.

[41] Duque I, Domínguez-Berjón MF, Cebrecos A, Prieto-Salceda M, Esnaola S, Calvo Sánchez M, Marí-Dell'Olmo M, en nombre del Grupo de Determinantes Sociales de la Salud‚ iniciativa contexto de la Sociedad Española de Epidemiología (2021). [Deprivation index by enumeration district in Spain, 2011]. Gac Sanit.

[42] Benchimol E, Smeeth L, Guttmann A, Harron K, Moher D, Petersen I, Sørensen HT, von Elm E, Langan SM, RECORD Working Committee (2015). The REporting of studies Conducted using Observational Routinely-collected health Data (RECORD) statement. PLoS Med.

[43] Calderón-Larrañaga A, Vetrano D, Onder G, Gimeno-Feliu L, Coscollar-Santaliestra C, Carfí A, Pisciotta MS, Angleman S, Melis RJF, Santoni G, Mangialasche F, Rizzuto D, Welmer AK, Bernabei R, Prados-Torres A, Marengoni A, Fratiglioni L (2017). Assessing and Measuring Chronic Multimorbidity in the Older Population: A Proposal for Its Operationalization. J Gerontol A Biol Sci Med Sci.

[44] Chavent M, Kuentz V, Labenne A, Liquet B, Saracco J PCAmixdata: Multivariate Analysis of Mixed Data. R Project.

[45] Karlis D, Saporta G, Spinakis A (2003). A Simple Rule for the Selection of Principal Components. Communications in Statistics - Theory and Methods.

[46] Calinski T, Harabasz J (1974). A dendrite method for cluster analysis. Comm. in Stats. - Theory & Methods.

[47] Bezdek JC (1973). Cluster Validity with Fuzzy Sets. Journal of Cybernetics.

[48] Carrasco-Ribelles L, Roso-Llorach A, Cabrera-Bean M, Costa-Garrido A, Zabaleta-Del-Olmo E, Toran-Monserrat P, Orfila Pernas F, Violán C (2022). Dynamics of multimorbidity and frailty, and their contribution to mortality, nursing home and home care need: A primary care cohort of 1 456 052 ageing people. EClinicalMedicine.

[49] Bauer S, Walter L, Ensrud K, Suskind A, Newman J, Ricke W, Liu TT, McVary KT, Covinsky K (2021). Assessment of Frailty and Association With Progression of Benign Prostatic Hyperplasia Symptoms and Serious Adverse Events Among Men Using Drug Therapy. JAMA Netw Open.

[50] Richardson K, Fox C, Maidment I, Steel N, Loke Y, Arthur A, Myint PK, Grossi CM, Mattishent K, Bennett K, Campbell NL, Boustani M, Robinson L, Brayne C, Matthews FE, Savva GM (2018). Anticholinergic drugs and risk of dementia: case-control study. BMJ.

[51] Forman D, Maurer M, Boyd C, Brindis R, Salive M, Horne F, Bell SP, Fulmer T, Reuben DB, Zieman S, Rich MW (2018). Multimorbidity in Older Adults With Cardiovascular Disease. J Am Coll Cardiol.

[52] Skou S, Mair F, Fortin M, Guthrie B, Nunes B, Miranda J, Boyd CM, Pati S, Mtenga S, Smith SM (2022). Multimorbidity. Nat Rev Dis Primers.

[53] Masoli J, Pilling L, Frayling T (2022). Genomics and multimorbidity. Age Ageing.

[54] Hanlon P, Nicholl BI, Jani BD, Lee D, McQueenie R, Mair FS (2018). Frailty and pre-frailty in middle-aged and older adults and its association with multimorbidity and mortality: a prospective analysis of 493 737 UK Biobank participants. The Lancet Public Health.

[55] Busija L, Lim K, Szoeke C, Sanders K, McCabe M (2019). Do replicable profiles of multimorbidity exist? Systematic review and synthesis. Eur J Epidemiol.

[56] Cezard G, McHale C, Sullivan F, Bowles J, Keenan K (2021). Studying trajectories of multimorbidity: a systematic scoping review of longitudinal approaches and evidence. BMJ Open.

[57] García-Gil MDM, Hermosilla E, Prieto-Alhambra D, Fina F, Rosell M, Ramos R, Rodriguez J, Williams T, Van Staa T, Bolíbar B (2011). Construction and validation of a scoring system for the selection of high-quality data in a Spanish population primary care database (SIDIAP). Inform Prim Care.

[58] Duda RO, Hart PE, Stork DG (2000). Pattern Classification, 2nd Edition.

[59] Cezard G, Sullivan F, Keenan K (2022). Understanding multimorbidity trajectories in Scotland using sequence analysis. Sci Rep.

[60] Howlett S, Rutenberg A, Rockwood K (2021). The degree of frailty as a translational measure of health in aging. Nat Aging.

[61] Data requests. SIDIAP.

