# Supplementary material for: Contribution of Frailty to Multimorbidity Patterns and Trajectories: Longitudinal Dynamic Cohort Study of Aging People
Source: JMIR Public Health Surveill. 2023 Jun 27;9:e45848. doi: 10.2196/45848 (PMC10365626; doi:10.2196/45848)
Supplement: Multimedia Appendix 2 [file publichealth_v9i1e45848_app2.pdf]

Multimedia Appendix 2. Supplementary methods

Smoking status and alcohol intake measurement

The information available up to the year of calculation on the start and end dates of smoking was used to determine which status had been predominant during follow-up. For example, to know the smoking status of a patient in 2010, we looked at the patient's clinical records up to 2010 and calculated the duration of each possible status (i.e. non smoker, ex-smoker or smoker), if available. The status that had been active for the longest time was assigned to the patient up to 2010. If the person had smoked for 25 years, until 2008, their predominant status in 2010 would still be "smoker". The status was assigned yearly, considering possible changes over time.

The criteria for classifying patients according to alcohol intake (i.e. non-drinker, low-risk drinker, high-risk drinker) are presented in Table S1. At the discretion of the physician, patients were asked about their alcohol use at regular intervals during visits to primary care. The software converts the drinks self-reported by the patient into grams to automate the calculation of the category in which they belong.

Table S1: Classification criteria for alcohol intake according to alcohol intake (grams) of alcohol ingested per week

	**Men**	**Women**
**Risk 0: non-drinker**	Non-drinker	Non-drinker
**Risk 1: low-risk drinker**	$<$280g of alcohol per week	$<$170g of alcohol per week
**Risk 2: high-risk drinker**	$<$280g of alcohol per week BUT<br>- is younger than 16 years old<br>- works with dangerous machinery<br>- is taking any drug that interferes with alcohol	$<$170g of alcohol per week BUT<br>- is pregnant<br>- is younger than 16 years old<br>- works with dangerous machinery<br>- is taking any drug that interferes with alcohol
	$\geq$280g of alcohol per week	$\geq$ 170g of alcohol per week
	Drinks sporadically $\geq$ 60g of alcohol over a short period of time, at least once a month.	Drinks sporadically $\geq$ 50g of alcohol over a short period of time, at least once a month.

Clustering analysis

This appendix describes the complete analysis pipeline followed to obtain the multimorbidity patterns and their trajectories (see Figure 1). In the following subsections, we denote the number of records as R, the number of quantitative features per record as N, and the number of categoric features as C.

Data

The dataset involved in the pattern identification involved one record per year of follow-up in included participant (mean 7.04 years, standard deviation 3.15 years) and 62 features. These features considered the 60 groups of multimorbidity conditions defined by the SNAC-K study [43] (see Table S2), one column with the number of frailty deficits as defined by eFRAGICAP [21], and one with the age of the individual related to each record. Since there is one record per person per year, each record had an associated age. The features related to chronic conditions were binary variables, indicating whether each individual had (1) or did not have (0) at least one active diagnosis from each of the 60 categories. On the other hand, the variables age and number of frailty deficits are continuous.

We first removed nine chronic conditions with a low ($< 2\%$) mean annual prevalence throughout the complete follow-up, including: chromosomal abnormalities, multiple sclerosis, schizophrenia, inflammatory bowel disease, chronic infectious diseases, haematological neoplasms, epilepsy, other respiratory diseases, and blood and blood-forming organ diseases. From there, two different datasets were defined. The first one included these 51 conditions plus age (*multimorbidity & age*), and the second one includes the 51 conditions plus the

Table S2: Prevalence of chronic conditions the year of inclusion and last year of follow-up.

Chronic condition	Start of follow-up	End of follow-up
**Allergy**	28,276 (1.94%)	41,967 (2.88%)
**Anaemia**	194,644 (13.4%)	379,051 (26.0%)
**Asthma**	62,233 (4.27%)	77,729 (5.34%)
**Atrial fibrillation**	85,842 (5.90%)	188,527 (12.9%)
**Autoimmune disease**	56,637 (3.89%)	86,879 (5.97%)
**Blindness, visual impairment**	33,469 (2.30%)	39,371 (2.70%)
**Blood and blood forming organ diseases**	17,723 (1.22%)	36,144 (2.48%)
**Bradycardia and conduction diseases**	24,840 (1.71%)	64,298 (4.42%)
**Cardiac valve diseases**	59,680 (4.10%)	114,926 (7.89%)
**Cataracts and other lens diseases**	156,399 (10.7%)	316,201 (21.7%)
**Cerebrovascular disease**	88,646 (6.09%)	98,009 (6.73%)
**Chromosomal abnormalities**	196 (0.01%)	228 (0.02%)
**Chronic infectious diseases**	10,523 (0.72%)	9,388 (0.64%)
**Chronic kidney disease**	261,530 (18.0%)	409,675 (28.1%)
**Chronic liver diseases**	34,141 (2.34%)	38,493 (2.64%)
**Chronic pancreas diseases, biliary tract and gallbladder diseases**	61,558 (4.23%)	91,135 (6.26%)
**Chronic ulcers**	14,489 (1.00%)	57,219 (3.93%)
**Colitis and related diseases**	224,016 (15.4%)	294,084 (20.2%)
**COPD, emphysema, chronic bronchitis**	143,141 (9.83%)	213,708 (14.7%)
**Deafness, hearing impairment**	115,029 (7.90%)	218,588 (15.0%)
**Dementia**	58,796 (4.04%)	196,523 (13.5%)
**Depression and mood diseases**	156,317 (10.7%)	227,816 (15.6%)
**Diabetes**	319,366 (21.9%)	389,799 (26.8%)
**Dorsopathies**	178,621 (12.3%)	276,689 (19.0%)
**Dyslipemia**	694,774 (47.7%)	745,472 (51.2%)
**Ear, nose, throat diseases**	59,966 (4.12%)	91,880 (6.31%)
**Epilepsy**	12,918 (0.89%)	21,390 (1.47%)
**Esophagus, stomach, duodenum diseases**	123,685 (8.49%)	231,040 (15.9%)
**Glaucoma**	108,159 (7.43%)	147,498 (10.1%)
**Heart failure**	79,389 (5.45%)	17,1917 (11.8%)
**Haematological neoplasms**	10,529 (0.72%)	21,466 (1.47%)
**Hypertension**	873,531 (60.0%)	976,500 (67.1%)
**Inflammatory arthropaties**	89,868 (6.17%)	149,349 (10.3%)
**Inflammatory Bowel disease**	8,576 (0.59%)	10,303 (0.71%)
**Ischemic heart disease**	123,230 (8.46%)	161,251 (11.1%)
**Migraine**	48,639 (3.34%)	57,656 (3.96%)
**Multiple sclerosis**	1,311 (0.09%)	1,388 (0.10%)
**Neurotic, stress-related and somatoform diseases**	204,001 (14.0%)	289,348 (19.9%)
**Obesity**	396,621 (27.2%)	484,648 (33.3%)
**Osteoarthritis and other degenerative joint diseases**	382,566 (26.3%)	527,195 (36.2%)
**Osteoporosis**	201,256 (13.8%)	217,626 (14.9%)
**Other cardiovascular diseases**	21,746 (1.49%)	45,117 (3.10%)
**Other digestive diseases**	26,155 (1.80%)	48,628 (3.34%)
**Other eye diseases**	57,267 (3.93%)	96,455 (6.62%)
**Other genitourinary diseases**	130,232 (8.94%)	198,151 (13.6%)
**Other metabolic diseases**	29,406 (2.02%)	51,502 (3.54%)
**Other musculoskeletal diseases**	189,383 (13.0%)	278,657 (19.1%)
**Other neurological diseases**	31,051 (2.13%)	61,780 (4.24%)
**Other psychiatric and behavioral diseases**	185,973 (12.8%)	135,999 (9.34%)
**Other respiratory diseases**	17,139 (1.18%)	42,491 (2.92%)
**Other skin diseases**	18,394 (1.26%)	35,904 (2.47%)
**Parkinson's and parkisonism**	22,870 (1.57%)	44,316 (3.04%)
**Peripheral neuropathy**	75,006 (5.15%)	117,328 (8.06%)
**Peripheral vascular disease**	36,673 (2.52%)	71,730 (4.93%)
**Prostate diseases**	204,,337 (14.0%)	281,837 (19.4%)
**Schizophrenia and delusional diseases**	7,604 (0.52%)	9,241 (0.63%)
**Sleep disorders**	129,879 (8.92%)	287,815 (19.8%)
**Solid neoplasms**	188,595 (13.0%)	341,862 (23.5%)
**Thyroid diseases**	133,523 (9.17%)	190,484 (13.1%)
**Venous and lymphatic diseases**	168,419 (11.6%)	250,749 (17.2%)

number of frailty deficits (*multimorbidity & frailty*). The process described below was applied separately to the two datasets.

Dimensionality reduction through PCAmix

Most clustering methods in general and fuzzy c-means in particular work with Euclidean distance. Therefore, it was necessary to transform our data, which included both categorical and numerical variables to generate a dataset amenable to applicaiton of this distance. To achieve this, we processed the dataset using PCAmix, which is a mixture of the well-known Principal Component Analysis (PCA) and Multiple Correspondence Analysis (MCA). The former works with numerical variables and the latter with categorical variables. Both methods are based on the analysis of the patterns of relationships between variables, generating a set of new variables from the original ones.

The PCAmix algorithm [44] applies a generalised singular value decomposition to the data, divided into two sub-matrices $Z = [Z_1, Z_2]$. Z_1 represents the standardised version of the quantitative information, with a dimension of R x N, while Z_2 represents the categorical information, with a dimension of R x C. When the generalised singular value decomposition is applied to Z, a transformation matrix F is computed to project the records in the dataset to a d dimensional space such as $d < N + C$ by selecting the eigenvectors associated with the first d largest singular values. d was chosen using the Karlis-Saporta-Spinaki rule [45], which recommends selecting the singular values that are greater than $1 + 2\sqrt{\frac{P+C-1}{R-1}}$. In our case, $d = 14$. Therefore, the resulting dataset once transformed had the dimension R x 14.

Soft clustering using fuzzy c-means

Clustering is based on the assignment of records to groups such that the records in the same cluster are as similar as possible, and as dissimilar as possible to

the records in other clusters. Fuzzy c-means is an unsupervised form of clustering in which records for each individual can be related to more than one cluster, or multimorbidity pattern, through a fuzzy membership. The dispersion of the membership probability is modulated by the hyperparameter m ($m > 1$), which measures the degree of fuzziness, and how much the clusters can overlap. The smaller m is, the more the fuzzy c-means will resemble the k-means, so each record is more likely to have a higher membership probability to a particular pattern. On the other hand, a higher m indicates that the membership probability is more widely distributed across all available patterns. The fuzzy c-means clustering process assigns a membership probability u_{ir} to each record r for $i = 1, ..., k$, where k is the number of clusters. Each cluster is assumed to define a multimorbidity pattern. u_{ir} then represents the membership probability of the record r of belonging to the cluster i. The similar/dissimilar properties in the fuzzy c-means are measured though a heuristic global cost function $J_m(U, V)$, which is the weighted sum of squared errors within groups:

$$J_m(\mathbf{U}, \mathbf{V}) = \sum_{r=1}^{R} \sum_{i=1}^{k} u_{ir}^m \|\mathbf{y_r} - \mathbf{v_i}\|^2;\ 1 < m < \infty \tag{1}$$

Once applied to the dataset, we obtained a membership matrix $U = u_{ir}$ of dimension Rxk, and a matrix of cluster centroids $V = v_1, ..., v_k$ of dimension dxk. These matrices are computed as follows:

$$u_{ir} = \frac{1}{\sum_{l=1}^{k} \left(\frac{\|\mathbf{y_r} - \mathbf{v_i}\|^2}{\|\mathbf{y_r} - \mathbf{v_l}\|^2} \right)^{\frac{1}{m-1}}};\ 1 \leq i \leq k;\ 1 \leq r \leq R \tag{2}$$

$$v_i = \frac{\sum_{r=1}^{R} u_{ir}^m\ y_r}{\sum_{r=1}^{R} u_{ir}^m};\ 1 \leq i \leq k \tag{3}$$

The norm $\|\mathbf{y_r} - \mathbf{v_i}\|^2$ in (1) defines a similarity measure between each record r and the cluster prototype or centroid v_i. m and k are the hyperparameters that have to be tuned to optimise the fuzzy c-means. The following values were considered: $k \in [2, 15]$, $m \in [1.1, 1.2, 1.4, 1.8]$. Calinski-Harabasz [46] index, partition entropy [47], the proportion of u_{ir} higher than 0.75, and the mean maximum u_{ir} were computed 100 times for each combination of k and m, in order to account for the random initialisation of the cluster centroids. Then, the values of the indices were averaged among the 100 repetitions and were compared to choose the best combination of hyperparameters. For each index, the best hyperparameter was the one from the clustering whose value was maximum except for partition entropy, which had to be minimum. This analysis pointed to the optimal value of $m = 1.1$, as it was the only one that showed any trend changes between ks, and $k \in [7, 12]$ for *multimorbidity & age* and $k \in [8, 13]$ for *multimorbidity & frailty* (see Figure S1). Once this range for k was found, $k = 11$ was chosen by the clinical team for both datasets after studying the characterisation of the multimorbidity patterns through the observed/expected ratio and the exclusivity of the conditions in each set of patterns.

Each of the 100 repetitions were performed in a random subsample of the population and consisted of the following process. The original dataset was divided randomly into a training (40%) and validation (60%) set. The training set was used to create the PCAmix transformer and to obtain the clustering model. Afterwards, both the transformer and the model were applied to the validation set. Then, the validation indices were calculated on both sets. The maximum difference observed in the prevalence of chronic conditions between this subset and the total population was 0.23%.

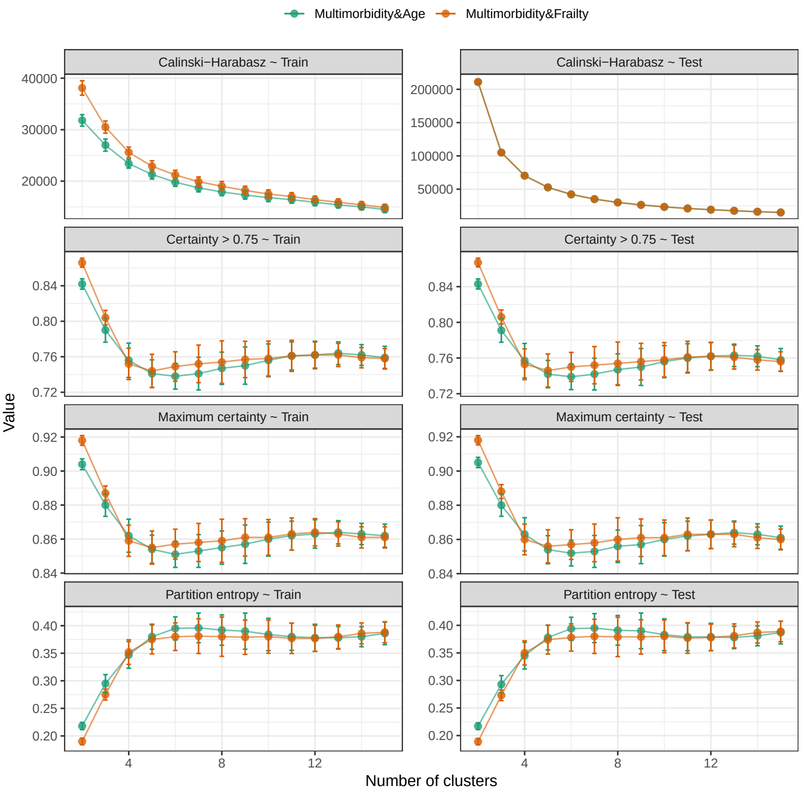

Fig. S1: Validation indices for both clustering models.
Only $m = 1.1$ results are shown, because we did not detect any trend change for the values for the rest of degrees of fuzziness that could be interpreted as a maximum or minimum. All indices but partition entropy should be maximised. Value represents the mean (standard deviation) among the 100 repetitions. Trends are very similar in both training and validation sets.

Calculation of the observed/expected ratio and exclusivity

The observed/expected (OE) ratio and the exclusivity ratio have been used to decide whether a chronic condition is representative in a given cluster.

The OE ratio is calculated by dividing the condition j prevalence in a cluster c (O) by the prevalence of the condition j in the overall population (E):

$$O_{c,j} = \frac{\sum individuals\ in\ cluster\ c\ with\ the\ condition\ j}{\sum individuals\ in\ the\ cluster\ c} \tag{4}$$

$$E_j = \frac{\sum individuals\ in\ the\ population\ with\ the\ condition\ j}{\sum individuals\ in\ the\ population} \tag{5}$$

Finally

$$OE_{c,j} = \frac{O_{c,j}}{E_j}$$

Exclusivity is calculated by dividing the individuals in cluster c with the condition j over the total number of individuals in the population with the condition j:

$$Exclusivity_{c,j} = \frac{\sum individuals\ in\ cluster\ c\ with\ the\ condition\ j}{\sum individuals\ in\ the\ population\ with\ the\ condition} \tag{6}$$

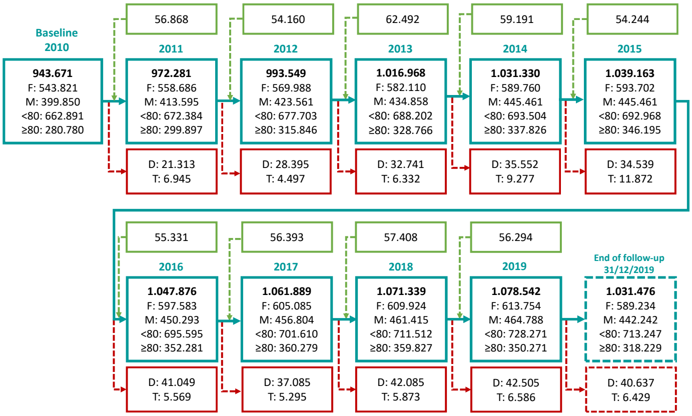

Fig. S2: Longitudinal flow chart of included persons during the study period (year 2010–2019).
Green arrows represent the inclusion of people reaching the age of 65, while red arrows represent the exclusion of persons previously included, either due to death (D) or transfer (T).
Note: F: Women; M: Men; <80: <80 years old; $\geq$80: 80 years or older.
