# Supplementary material for: Contribution of Frailty to Multimorbidity Patterns and Trajectories: Longitudinal Dynamic Cohort Study of Aging People
Source: JMIR Public Health Surveill. 2023 Jun 27;9:e45848. doi: 10.2196/45848 (PMC10365626; doi:10.2196/45848)
Supplement: Multimedia Appendix 3 [file publichealth_v9i1e45848_app3.docx]

**Multimedia Appendix 3.** Longitudinal flowchart of included persons during the study period (year 2010-2019).


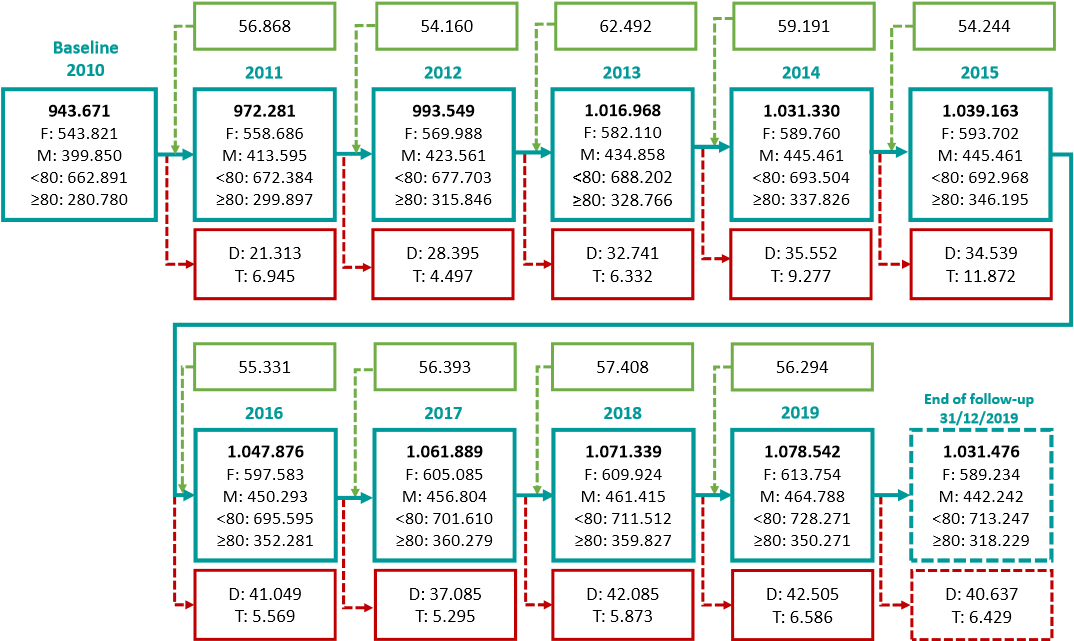


Green arrows represent the inclusion of people reaching the age of 65, while red arrows represent the exclusion of persons previously included, either due to death (D) or transfer (T).

*Note*: F: Women; M: Men; *<* 80: *<*80 years old; ≥80: 80 years or older.
