# Supplementary material for: Contribution of Frailty to Multimorbidity Patterns and Trajectories: Longitudinal Dynamic Cohort Study of Aging People
Source: JMIR Public Health Surveill. 2023 Jun 27;9:e45848. doi: 10.2196/45848 (PMC10365626; doi:10.2196/45848)
Supplement: Multimedia Appendix 6 [file publichealth_v9i1e45848_app6.docx]

**Multimedia Appendix 6.** Statistics of the trajectories according to follow-up time.

A


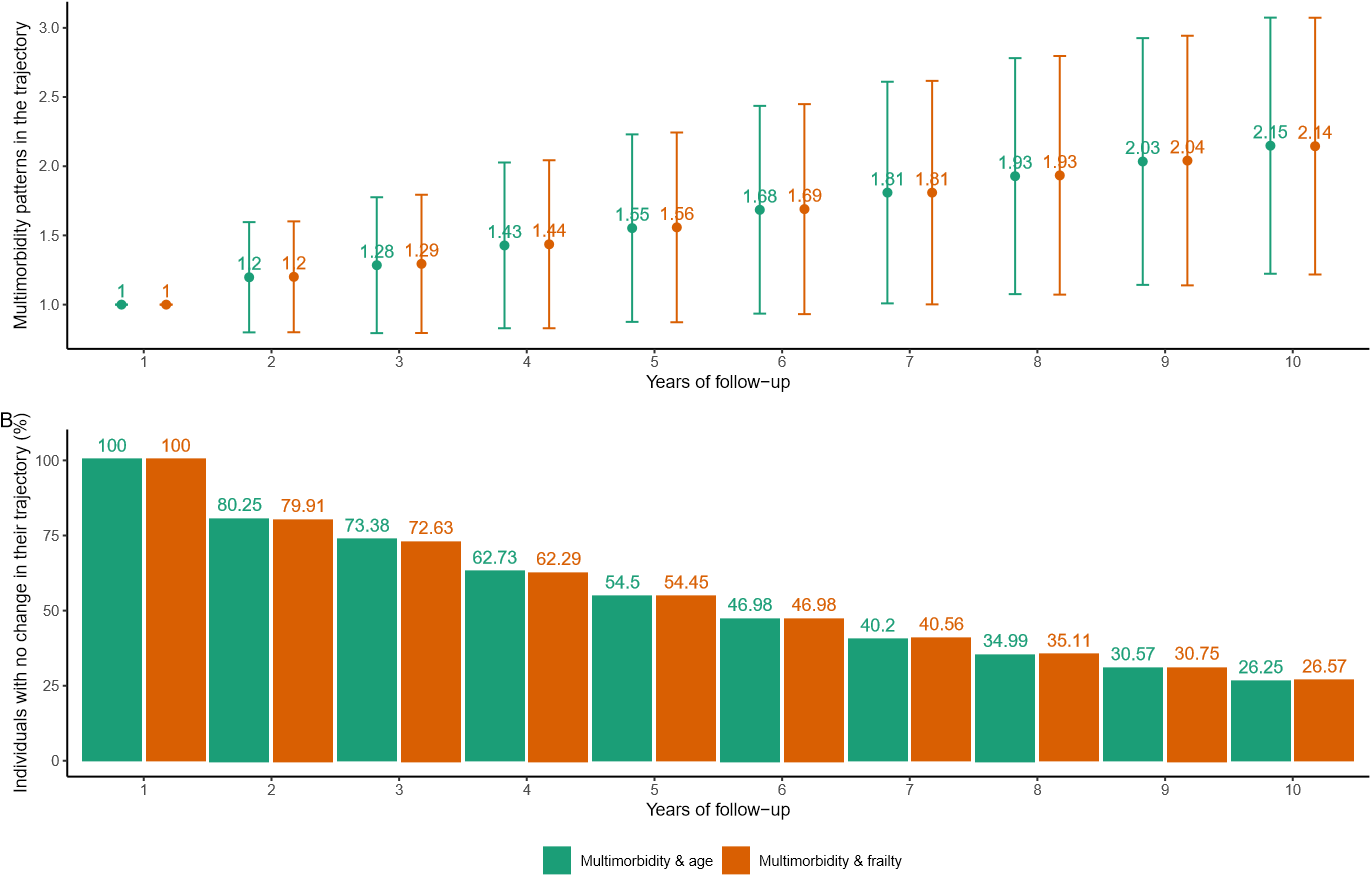


(A) The mean (± standard deviation) number of patterns defining individuals’ trajectories according to the duration of their follow-up. (B) The proportion of individuals which remained in the same pattern throughout their follow-up.
