# Supplementary material for: Contribution of Frailty to Multimorbidity Patterns and Trajectories: Longitudinal Dynamic Cohort Study of Aging People
Source: JMIR Public Health Surveill. 2023 Jun 27;9:e45848. doi: 10.2196/45848 (PMC10365626; doi:10.2196/45848)
Supplement: Multimedia Appendix 7 [file publichealth_v9i1e45848_app7.docx]

**Multimedia Appendix 7.** Trajectories of 50 random participants aged 65, 75, or 85 years in 2010 (baseline year) according to *multimorbidity & age* patterns with *k* = 11.

A


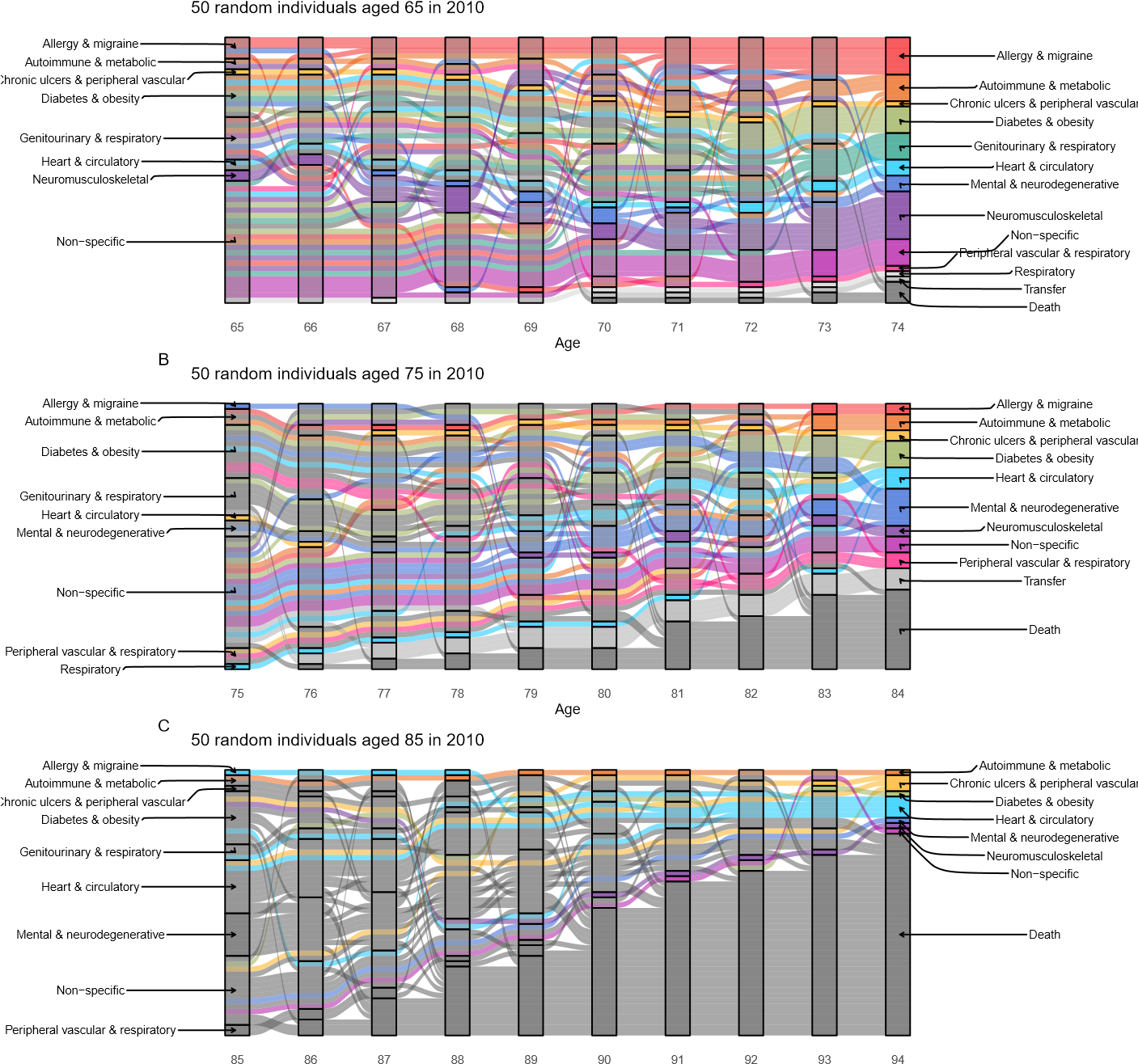


Age

Each line represents the evolution of a person.
